# Genome Architecture Facilitates Phenotypic Plasticity in the Honeybee (*Apis mellifera*)

**DOI:** 10.1093/molbev/msaa057

**Published:** 2020-04-05

**Authors:** Elizabeth J Duncan, Megan P Leask, Peter K Dearden

**Affiliations:** m1 Genomics Aotearoa and Biochemistry Department, University of Otago, Dunedin, New Zealand; m2 School of Biology, Faculty of Biological Sciences, University of Leeds, Leeds, United Kingdom

**Keywords:** phenotypic plasticity, reproductive constraint, ChIP-seq, H3K27me3, genomic regulatory domain, gene cluster, RNA-seq, polycomb, honeybee, evolution of gene expression, *Apis mellifera*

## Abstract

Phenotypic plasticity, the ability of an organism to alter its phenotype in response to an environmental cue, facilitates rapid adaptation to changing environments. Plastic changes in morphology and behavior are underpinned by widespread gene expression changes. However, it is unknown if, or how, genomes are structured to ensure these robust responses. Here, we use repression of honeybee worker ovaries as a model of plasticity. We show that the honeybee genome is structured with respect to plasticity; genes that respond to an environmental trigger are colocated in the honeybee genome in a series of gene clusters, many of which have been assembled in the last 80 My during the evolution of the Apidae. These clusters are marked by histone modifications that prefigure the gene expression changes that occur as the ovary activates, suggesting that these genomic regions are poised to respond plastically. That the linear sequence of the honeybee genome is organized to coordinate widespread gene expression changes in response to environmental influences and that the chromatin organization in these regions is prefigured to respond to these influences is perhaps unexpected and has implications for other examples of plasticity in physiology, evolution, and human disease.

## Introduction

Phenotypic plasticity allows organisms to respond to their environment by dramatically changing their physiology and behavior without altering their underlying genotype (Pigliucci 2001; Nijhout 2003; West-Eberhard 2005). Examples of phenotypic plasticity include changes in the morphology of the crustacean *Daphnia* due to predation (Laforsch and Tollrian 2004), or male horn length in species of horned beetles (Moczek 1998). Significant changes in shape, color, or form imply that global coordinated control of transcription and epigenetic regulation of the genome are required to change phenotype in response to environmental factors (Kucharski et al. 2008; Brakefield and Frankino 2009). Most animals have some degree of plasticity encoded in their genomes, as all have an adaptive need to modify their biology in response to environmental change (Moczek 2010; Beldade 2019). How globally coordinated changes in gene expression in response to environmental stimuli are regulated, however, remains largely unknown. To determine the genomic and epigenetic systems that establish and maintain phenotypic plasticity, we used a tractable and reliable model system: the honeybee *Apis mellifera*.

Honeybees exhibit remarkable examples of phenotypic plasticity: responding dramatically and predictably to environmental cues to generate distinct phenotypes (Winston 1991). A nutritional stimulus, royal jelly, fed to young female larvae is sufficient to trigger queen development and the absence of this stimulus leads to the development of worker bees (Huber 1821; Winston 1991). During larval development worker ovaries partially degenerate (Hartfelder and Steinbrück 1997), however, adult workers retain some reproductive capacity (Jay 1968; Velthuis 1970; Oldroyd et al. 2001). In a honeybee colony, the dominant female, the queen, carries out the majority of reproduction. Such reproductive division of labor is the cornerstone of eusociality. The fact that the worker caste retains some ability to reproduce generates a source of conflict in social insect colonies (Ratnieks et al. 2006) and mechanisms have evolved to prevent reproduction in the worker caste (Khila and Abouheif 2008, 2010; Duncan et al. 2016; Ronai et al. 2016). In honeybee workers, this reproductive capacity is plastic and is responsive to pheromones produced by brood and the queen, including queen mandibular pheromone (QMP) (Butler and Fairey 1963; Hoover et al. 2003), which acts to keep adult worker ovaries quiescent in the presence of the queen. QMP, which is a blend of five major chemicals (Slessor et al. 1988), is highly derived and the components of QMP share little chemical similarity with the majority of known hymenopteran queen pheromones. Commonly, these hymenopteran queen pheromones are derived from cuticular hydrocarbons (Van Oystaeyen et al. 2014; Princen et al. 2019) and it has been hypothesized that these pheromones have evolved from by-products of ovarian activity, sex pheromones, or oviposition deterring pheromones (reviewed by Oi et al. [2015]).

If the queen is lost from a honeybee hive the workers respond to this environmental cue and develop active ovaries (Hess 1942; Jay 1968; Velthuis 1970). During plastic activation of the worker ovary, the tissue is completely remodeled, developing differentiated cell types, producing oocytes (Velthuis 1970), switching on vitellogenesis (Koywiwattrakul and Sittipraneed 2009), and finally producing and laying mature haploid eggs (Hess 1942). Here, we compare gene expression and chromatin modifications in ovaries of queen-right workers (small quiescent ovaries) and queen-less workers with ovaries undergoing active oogenesis. We use this example of plasticity as a model to investigate the coordination of gene regulation underlying plastic responses.

We have previously shown that Notch signaling in the germarium of worker bee ovaries is a key molecular controller required for the establishment of worker reproduction (Duncan et al. 2016). Notch signaling is active in the germarium, the region of the ovary where oocytes are specified, of queen-right worker bees. Loss of the queen and her pheromone (QMP) is associated with degradation of the Notch receptor and loss of Notch signaling in this key region, and consequently, plastic activation of oogenesis. Importantly, Notch signaling has a functional role in inhibiting reproduction; treating bees with a chemical inhibitor of Notch signaling (N-[N-(3,5-Difluorophenacetyl)-L-alanyl]-S-phenylglycine t-butyl ester) increased the proportion of bees with active ovaries even in the presence of QMP—demonstrating that Notch signaling is a key regulator of reproductive plasticity in the honeybee (Duncan et al. 2016). Notch signaling is a conserved cell-signaling pathway with the potential to coordinate global gene expression, mediated by regulation of histone modifiers (Bray et al. 2005). Histone modifiers and the modifications that they create have been implicated in plasticity (Duncan et al. 2014) including diapause, metamorphosis, longevity, and developmental polyphenisms in insects (Simola et al. 2013, 2016; Wojciechowski et al. 2018). Differences in the chromatin landscape also underpin gene expression changes associated with caste specification during larval development in the honeybee (Wojciechowski et al. 2018).

Phenotypic plasticity has important consequences for adaptive evolution (West-Eberhard 2005; Fusco and Minelli 2010) and human health (Bateson et al. 2004; Gluckman et al. 2011). It is crucial that we understand the molecular and epigenetic mechanisms that control phenotypic plasticity. Here, we report genome-wide analyses of phenotypic plasticity in the ovaries of reproductively repressed queen-right and reproductively active queen-less worker bees identifying structural and epigenetic features of the genome that facilitate plastic responses to the environment.

## Results

### Notch, Polycomb, and Honeybee Ovary Plasticity

**Fig. 1.**
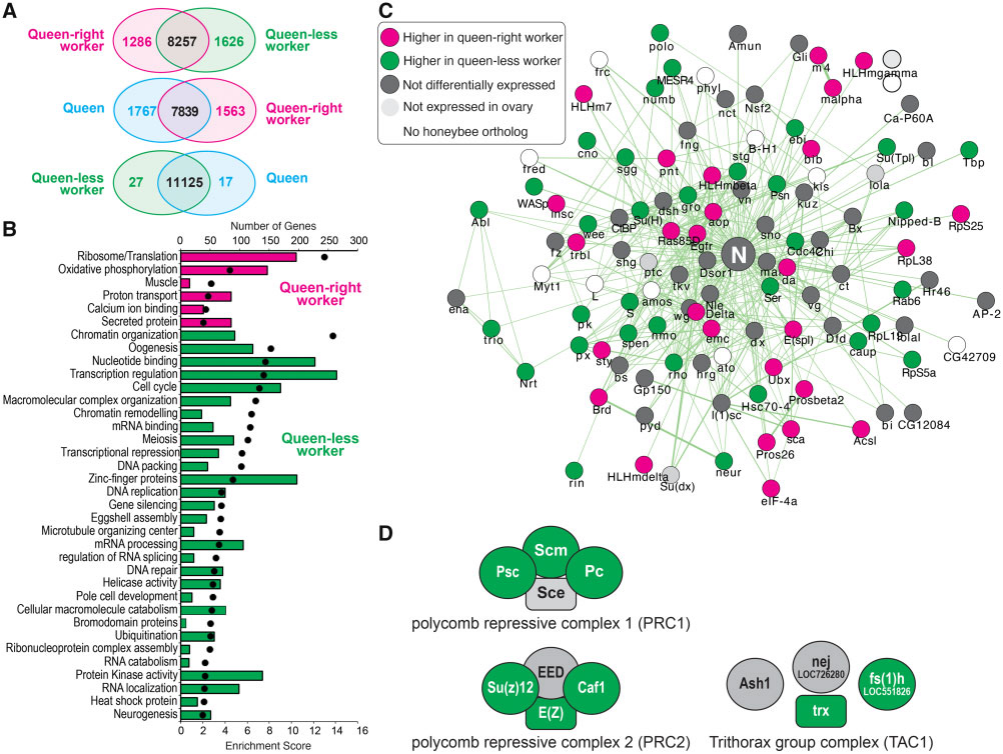
RNA-seq analysis of gene expression in the ovaries of queen-right workers (reproductively inactive), queen-less workers, and queens (both reproductively active). (*A*) Venn diagrams illustrate the number of genes differentially expressed in each pairwise comparison of the RNA-seq data (FDR corrected *P* value ≤ 0.01). (*B*) Gene ontology categories significantly enriched in queen-right ovaries (magenta) and queen-less ovaries (green), bars indicate number of genes, whereas spots indicate enrichment score. (*C*) Gene interaction network depicting physical and genetic interactions with the Notch signaling receptor in *Drosophila melanogaster*. Nodes are colored according to expression in honeybee ovaries (magenta, higher expression in queen-right; green, higher expression in queen-less; dark gray, not differentially expressed; light gray, not expressed in ovary; white, no known honeybee ortholog of the *Drosophila* gene). (*D*) Differential expression of polycomb group (PRC1 and PRC2) and trithorax group proteins (TAC1) in the honeybee ovary. Green, higher expression in queen-less (reproductively active) workers; gray, not differentially expressed.

RNA-seq was used to identify genes that were differentially expressed between queen bee ovaries, worker bee ovaries in the presence of a queen (queen-right) and queen-less worker bee ovaries producing mature eggs (fig. 1). In response to the loss of the queen and her pheromone (QMP), reproductively repressed queen-right worker ovaries are transformed into a tissue with similar gene expression to queen ovaries (fig. 1*A*), with 2,912 genes differentially expressed between queen-right and queen-less worker ovaries and only 44 genes expressed differentially between queen-less worker ovaries and queens. Genes more highly expressed in queen-right worker ovaries are enriched for gene ontology terms associated with energy production and protein translation (fig. 1*B*). Genes more highly expressed in queen-less worker ovaries are enriched for gene ontology categories that include chromatin organization, chromatin remodeling, oogenesis, and neurogenesis (fig. 1*B*).

Network analysis of genes in the “neurogenesis” gene ontology category indicated that the E(spl)-C genes, which are key transcriptional targets of Notch signaling (Jennings et al. 1994; Duncan and Dearden 2010), nucleated this network. Systematic analysis of the expression of genes associated with *Drosophila* Notch signaling revealed that reproductive plasticity in the honeybee is associated with changes in expression of a large number of genes in this network (fig. 1*C*). This is consistent with our previous functional studies indicating that Notch signaling is a key modulator of reproductive plasticity in the honeybee (Duncan et al. 2016). Chromatin modification enzymes were also identified as responding to ovary activation (fig. 1*B*), in particular, genes that encode the polycomb repression complex (PRC) 1 and 2 and the trithorax acetylation complex (TAC) (fig. 1*D*).

In total, 2,912 genes are differentially expressed between repressed and active ovaries. This constitutes 26.1% of all the genes identified in the honeybee genome and 32.9% of the genes expressed in the honeybee ovary. These global changes in gene expression, together with differential expression of genes involved in chromatin remodeling, implies that the plastic activation of worker honeybee ovaries involves complex coordinated changes in gene expression across the whole genome.

### Ovarian Plasticity and Genome Organization

We hypothesized that genes responding plastically to ovary activation may be clustered together on the chromosomes, as being clustered together may more efficiently facilitate coordinated expression (as seen for the Hox [Pace et al. 2016], runt [Duncan et al. 2008], and E(spl)-C [Duncan and Dearden 2010] complexes). Such clustering might be expected given muscle-expressed genes appear clustered in *Caenorhabditis elegans* (Roy et al. 2002), testis-expressed genes show clustering in *Drosophila* (Boutanaev et al. 2002), and studies of genes in primate genomes indicate that evolutionary changes in the expression of a gene often affects the expression of its neighbors (Ghanbarian and Hurst 2015). By comparing genes that are expressed plastically between queen-right, queen-less, and queen ovaries, we discovered that genes that are differentially expressed are significantly more often found in clusters than would be expected by chance (table 1). We determined that 35% of the 2,912 genes differentially expressed between queen-right and queen-less worker ovaries are found in physical clusters ranging in size from three to nine genes in the honeybee genome (supplementary tables 1 and 2 and fig. 1, Supplementary Material online). This implies that the organization of genes on honeybee chromosomes is both functionally important for, and potentially influenced by, plasticity and ovary activation. Given this finding, we examined other RNA-seq data sets from honeybees for evidence of physical clustering. Such clustering is evident, but less prevalent, in other RNA-seq data sets (supplementary table 5, Supplementary Material online) and only a few of these clusters (0.016%) are shared between data sets (supplementary fig. 2, Supplementary Material online). This implies that the genome of the honeybee is nonrandomly organized with respect to a range of gene expression responses, including plasticity.

**Table 1.** Summary of Cluster Based (CROC) Analysis of Differentially Expressed (DE) Genes.

DE Gene List	Comparison	Number of DE Genes	*Gene-Based Analysis* ^a^		*Window-Based Analysis* ^b^	
			Clusters	Significance	Clusters	Significance
Queen-right worker	Queen-less worker	1,286	28	0.0399	30	0.0076
Queen-less worker	Queen-right worker	1,626	32	0.0049	46	0.0003
Queen-right worker	Queen	1,563	27	0.0407	34	0.0121
Queen	Queen-right worker	1,767	31	0.0061	49	0.0001
Queen-less worker	Queen	27	1	0.0003	1	0.0013
Queen	Queen-less worker	17	0	n/a	0	n/a

a Clusters were defined based on three differentially expressed genes occurring within a group of five genes on the honeybee chromosome (irrespective of the size of genes).

b Clusters were defined based on detecting three differentially expressed genes within a series of 50-kb windows, with an offset of 1 kb.

### Evolution of Honeybee “Plasticity Clusters”

As plasticity appears to be one of the factors that may have shaped the organization of the honeybee genome by producing clusters of coregulated genes, we asked if the clusters identified (table 1) as responding to the presence or absence of the queen were ancestral features of hymenopteran genomes, coopted into ovary activation, or new clusters of genes assembled during the evolution of honeybees and ovary activation. Comparing protein sequence similarity between members of each cluster, we determined whether genes within these clusters were likely to have arisen by gene duplication or whether they are unrelated (at the sequence level) and therefore unlikely to have arisen as a result of gene duplication. We conclude that genes within these clusters have not generally evolved by gene duplication, unlike the Hox (Pace et al. 2016) and runt gene clusters (Duncan et al. 2008). Instead, the majority of these clusters contain at least two classes of genes unrelated at the sequence level (supplementary tables 1 and 2, Supplementary Material online) similar to the E(spl)-C (Duncan and Dearden 2010).

**Fig. 2.**
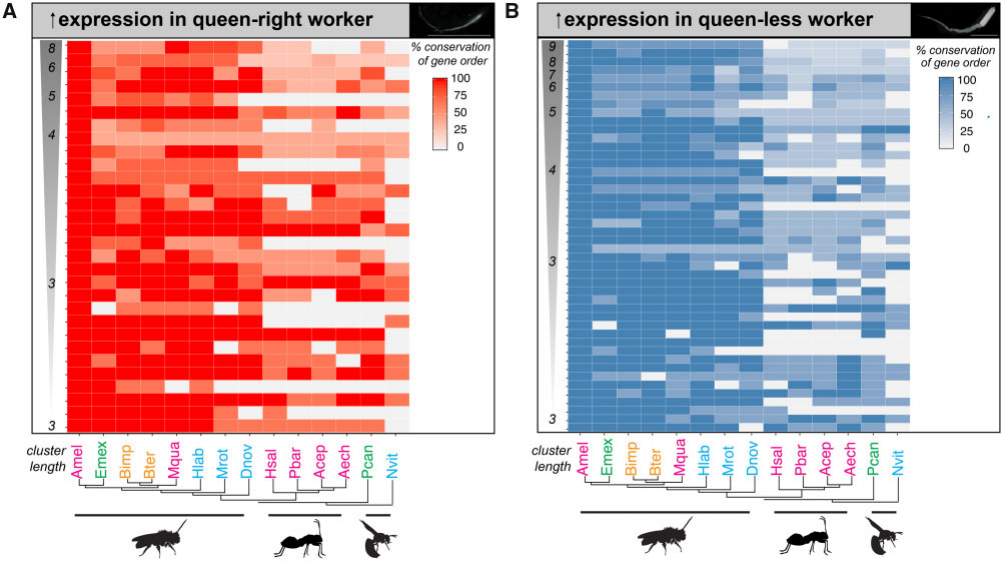
Evolutionary histories of coordinately regulated clusters of differentially expressed genes within the Hymenoptera. (*A*) Heatmap depicting the conservation of “Queen responsive” clusters of coordinately regulated genes that are more highly expressed in queen-right (reproductively inactive) worker ovaries. (*B*) Heatmap depicting the conservation of “plasticity responsive” clusters of coordinately regulated genes that are more highly expressed in queen-less (reproductively active) worker ovaries. Intensity of the color depicts level of conservation along a continuous scale as indicated by the key. Clusters are ordered along the *y*-axis by the number of genes contained within the cluster the largest cluster the top of the heatmap. Phylogeny of the species is depicted on the *y*-axis (Peters et al. 2017). Social complexity is indicated by color of the species names (cyan, ancestrally solitary; green, facultative simple eusociality; orange, obligate simple eusociality; magenta, obligate complex eusociality). Species names are Amel, *Apis mellifera*; Emex, *Eufriesea mexicana*; Bimp, *Bombus impatiens*; Bter, *Bombus terrestris*; Mqua, *Melipona quadrifasciata*; Hlab, *Habropoda laboriosa*; Mrot, *Megachile rotundata*; Dnov, *Dufourea novaeangliae*; Hsal, *Harpegnathos saltator*; Pbar, *Pogonomyrmex barbatus*; Acep, *Atta cephalotes*; Aech, *Acromyrmex echinatior*; Pcan, *Polistes canadensis*; Nvit, *Nasonia vitripennis*.

Our analysis (fig. 2) indicates that there is a mixture of evolutionary histories for these gene clusters. Overall, 40% of these clusters have been assembled over the last 80 My (Peters et al. 2017) specifically in Apidae (supplementary fig. 3, Supplementary Material online, an example cluster demonstrating the evolution of one of these clusters is shown in supplementary fig. 4, Supplementary Material online). Intriguingly, clusters of genes more highly expressed in repressed queen-right workers as compared with queen-less workers (“Queen responsive clusters”) appear to have longer evolutionary histories. In total, 43% of these clusters are conserved (defined by conservation of gene order of 75% of genes within a cluster) in *Nasonia vitripennis*, which is 235 My diverged from honeybee (Peters et al. 2017) (fig. 2*A* and supplementary fig. 3, Supplementary Material online). In contrast, only 24% of clusters of genes more highly expressed in queen-less worker ovaries as compared with repressed queen-right workers (“Plasticity responsive clusters”) show 75% conservation of gene order in *N. vitripennis* (fig. 2*B* and supplementary fig. 3, Supplementary Material online).

### PRC2 Activity Changes during Plasticity

**Fig. 3.**
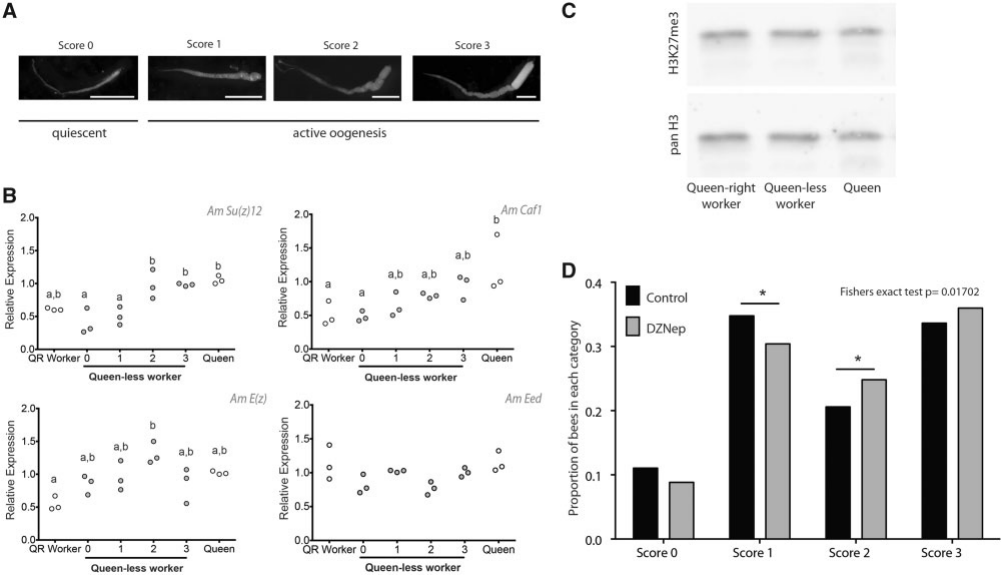
Expression of genes of the PRC2 and localization of H3K27me3 in the honeybee ovary. (*A*) Ovary activity in queen-less worker bees is scored on a modified Hess scale. (*B*) RT-qPCR of genes encoding proteins of the PRC2. Target gene expression is measured relative to *mRPL44* and *Rpn2*, which are stably expressed in honeybee ovaries (Duncan et al. 2016). Gene expression was measured in three biological replicates each consisting of ovaries from multiple individuals: queen (*n* = 3), queen-right worker (*n* = 20), and queen-less workers (score 0, *n* ∼ 20; score 1, *n* ∼ 20; score 2, *n* ∼ 10; and score 3, *n* ∼ 10). Differences in gene expression were assessed using a general linear model ANOVA with a Tukey post hoc test and 95% confidence interval. Samples that do not share letters are statistically significantly different with a *P* value <0.05. (*C*) Western blot analysis of ovary histone extracts for enrichment of H3K27me3 in queen, queen-right worker, and queen-less worker ovary (full blot: supplementary fig. 5, Supplementary Material online). (*D*) Inhibition of histone methylation using DZNep enhances ovary activity in honeybee workers. Proportion of bees scored as reproductively inactive (score, 0), and degrees of reproductively active (score 1–3) following treatment of newly emerged bees for 10 days with 50 μM DZNep (*n* = 524) or control (*n* = 532). Experiments were performed in triplicate on two separate occasions.

One explanation for the clusters of coregulated genes is that these may be chromatin domains (Dixon et al. 2016) in which the gene cluster is regulated by chromatin modifiers over a broad genomic area. Histone modifications, posttranslational modifications of key components of nucleosomes, appear to regulate the accessibility of genes to transcription over broad areas of the genome (Dixon et al. 2016). Our gene expression analysis highlighted differences in expression of genes encoding components of the PRC2 protein complex during ovary activation (specifically, Su(Z)12, E(z), and caf1, fig. 1*D*). PRC2 acts as a histone methyltransferase involved in targeting regions of the genome for trimethylation of histone H3 (H3K27me3). Trimethylation of histone H3 (H3K27me3) is associated with repressive heterochromatin in *Drosophila* and other species (Filion et al. 2010). Little is known about its function in the honeybee genome, but it is likely involved in changes in gene expression across broad regions of the genome (Entrevan et al. 2016). Because of this possibility, we examined in more detail the PRC2 components in queen-right and queen-less worker ovaries (fig. 3).

Genes that encode specific components of the PRC2 complex change expression during ovary activation (fig. 3*A* and *B*). *E(z)*, the key methyltransferase in the PRC2 complex, has higher expression in ovaries scored as 2 (on the modified Hess scale [Hess 1942; Duncan et al. 2016], fig. 3*A* and *B*) compared with queen-right workers. However, overall levels of the H3K27me3 mark in ovary tissues do not vary (fig. 3*C* and supplementary fig. 5, Supplementary Material online).

Our results indicate that PRC2 and H3K27me3 may, in part, mediate phenotypic plasticity, and to functionally test this hypothesis, we blocked the activity of the PRC2 using the inhibitor 3-Deazaneplanocin A (DZNep). DZNep is an *S*-adenosyl-l homocysteine hydrolase inhibitor that depletes E(z) (Tan et al. 2007) and has been used in insects to reduce levels of H3K27me3 (Lu et al. 2013). Treating newly emerged worker bees with this inhibitor led to a significant increase in ovary activity compared with controls (fig. 3*D* and supplementary fig. 6, Supplementary Material online). The effect of DZNep was smaller than reported for blocking Notch cell signaling (Duncan et al. 2016) and indicated a significant shift from stage 1 to stage 2 ovaries (fig. 3*D*), later stages of ovary activation, rather than the early changes caused by blocking Notch (Duncan et al. 2016). This may indicate a role for PRC2 in determination and maturation of the oocytes rather than specification.

Given the changes in expression of genes encoding PRC2 components and the impact of blocking H3K27me3, we hypothesized that differences between repressed (queen-right) and active (queen-less) ovaries might be reflected in the placement of H3K27me3 marks across the genome, and that these may be associated with the clusters of coregulated genes.

### H3K27me3 Marks Clusters of Plasticity Genes

**Fig. 4.**
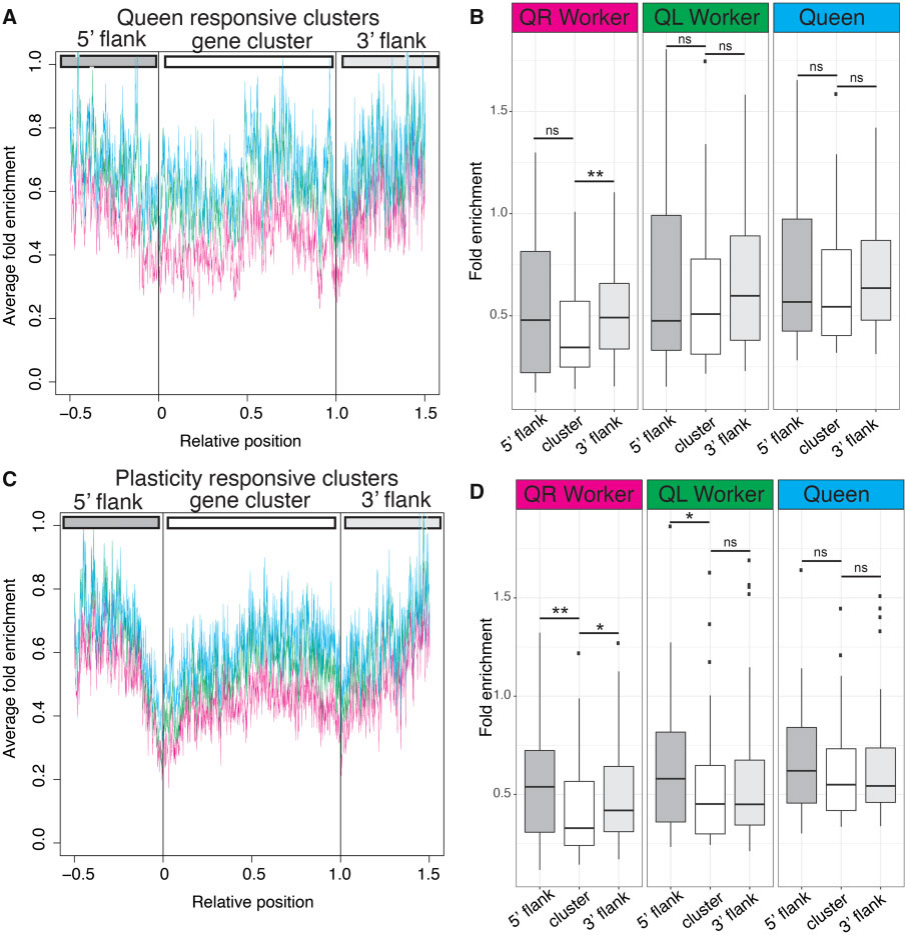
H3K27me3 stably defines “plasticity responsive” gene clusters, prefiguring changes in gene expression. (*A*) Average H3K27me3 enrichment across gene clusters more highly expressed in queen-right workers (Queen responsive clusters). Cyan is queen H3K27me3 enrichment, green is from queen-less workers, and magenta is from queen-right workers. (*B*) Boxplot illustrating H3K27me3 enrichment across gene clusters more highly expressed in queen-right workers (Queen responsive clusters). Only the 3′ flank of the clusters is significantly enriched for H3K27me3 in queen-right worker ovaries (Wilcoxon rank sum test, *P < 0.05, **P < 0.01, ns = not significant), showing that H3K27me3 marks are dynamic with respect to the presence of a queen and her pheromone. (*C*) Average H3K27me3 enrichment across gene clusters more highly expressed in queen-less worker ovaries (Plasticity responsive gene clusters) showing a decrease in H3K27me3 enrichment demarking both the 5′ and 3′ edges of the cluster. (*D*) Boxplot illustrating H3K27me3 enrichment across gene clusters more highly expressed in queen-less workers (Plasticity responsive clusters). In this case, we see that H3K27me3 marks are relatively stable, with both the 5′ and 3′ flanks of the cluster showing significant enrichment for H3K27me3 relative to the body of the cluster (Wilcoxon rank sum test, *P < 0.05, **P < 0.01, ns = not significant) even though the expression of these genes is low in queen-right workers. Boxplot whiskers indicate minimum and maximum, the box is defined by 25th percentile, median, and 75th percentile. Outliers, data points outside 1.5 times the interquartile range above the upper quartile and below the lower quartile, are shown as individual data points.

We compared average H3K27me3 enrichment in each of the three ovary states (queen, queen-right workers, and queen-less workers) across the length of all “Queen responsive clusters” (fig. 4*A* and *B*) or “Plasticity responsive clusters” (fig. 4*C* and *D*) as a way to understand how epigenetic marks relate to the gene clusters we identify, and to determine if these marks change when transitioning from a queen-right to a queen-less state.

Clusters of genes we identify as being more highly expressed in queen-right worker bees (fig. 2*A* and supplementary table 1, Supplementary Material online) are likely involved in repression of oogenesis in response to the queen as their expression is modulated by the presence of the queen and her pheromone (QMP). These “Queen responsive clusters” are characterized by variable levels of H3K27me3 across the cluster, with a slight decrease at the borders of the cluster (fig. 4*A*) and slightly higher levels of H3K27me3 on regions of DNA flanking the gene clusters (fig. 4*A*). Significantly higher levels of H3K27me3 are observed for the 3′ flank region compared with levels of H3K27me3 within the gene cluster, but only in queen-right workers (fig. 4*B*). This pattern is not seen in queen-less worker ovaries, implying that higher levels of H3K27me3 in the chromatin at the 3′ flank region are transient and associated with repression of ovary activity by the queen and her pheromone (QMP) (fig. 4*A* and *B*).

Clusters of genes that are more highly expressed in queen-less worker ovaries are genes that are expressed plastically in response to the loss of the queen (fig. 2*B* and supplementary table 2, Supplementary Material online). In contrast to the “Queen responsive clusters” detailed above, these “Plasticity responsive clusters” show a more marked pattern of H3K27me3 enrichment across the body of the gene cluster and in the flanking regions (fig. 4*C*), with the boundaries of the cluster demarked by low levels of H3K37me3 enrichment (boundaries of the cluster are indicated by the 0 and 1 relative positions). Quantification of the enrichment of H3K27me3 across the flanking regions and cluster body (fig. 4*D*) indicates that these clusters are characterized by higher levels of H3K27me3 on regions of DNA flanking the gene clusters compared with levels of H3K27me3 within the cluster, particularly on the 5′ flank. However, unlike the “Queen responsive clusters,” this pattern is stable, as these gene regions are marked in a similar way in queen-right workers, but not queens (fig. 4*C* and *D*). This pattern of low H3K27me3 at the boundaries of the clusters and higher levels on the flanks than across the cluster body is not present in individual genes in each cluster (supplemental fig. 7, Supplementary Material online), indicating that this pattern is not an artifact of cluster definition.

“Plasticity responsive clusters” contain genes that will increase expression in queen-less worker ovaries when the queen, and her pheromone, is removed. Our analysis indicates that these clusters are marked by reductions in H3K27me3 at the boundary of clusters in a similar way in both queen-right and queen-less worker ovaries. This implies that these genomic regions are prefigured in queen-right worker ovaries to respond to the loss of the queen and her pheromone, as this pattern is seen *before* ovary activation occurs.

### Differential Enrichment of H3K27me3 Is Associated with Notch Signaling

Although whole-genome levels of H3K27me3 do not change between repressed and active worker ovaries (fig. 3*C* and supplementary fig. 8*A* and *B*, Supplementary Material online), and plasticity-related clusters are stably marked with H3K27me3, we asked if other genomic regions were differentially marked with H3K27me3 with respect to plasticity (supplementary text and fig. 8, Supplementary Material online). As H3K27me3 marks often appear in broad peaks that may be difficult to detect using peak calling software (Pauler et al. 2009), we also used a sliding window approach (Shen et al. 2013) to identify regions of differential enrichment (supplementary fig. 8, Supplementary Material online).

Differentially enriched peaks (supplementary fig. 8*C*, Supplementary Material online) were associated with different genomic features than differentially enriched windows (supplementary fig. 8*D*, Supplementary Material online), which may reflect the fact that sharper regions of enrichment, more likely to be called as peaks, are associated with particular genomic features, including promoter regions (supplementary fig. 8*A*, Supplementary Material online). H3K27me3 marks in promoter regions have been identified as marking “poised promoters” (Rada-Iglesias et al. 2011), prefiguring rapid responses to changes in genome regulation. Of the 696 genes that have H3K27me3 peaks in their promoter regions, a subset of these genes (*n* = 286 genes) have altered expression, indicating that these may be poised promoters that respond to the loss of the queen and her pheromone (supplementary fig. 8*C*, Supplementary Material online). These genes are enriched for gene ontology terms associated with neurogenesis and nervous system development and include genes involved in Notch signaling.

**Fig. 5.**
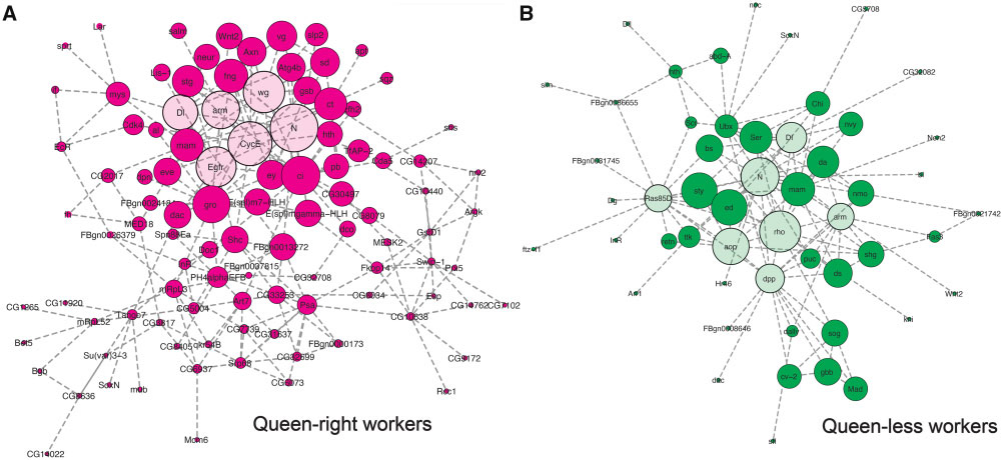
Network analysis identifies key hubs genes associated with differential enrichment of H3K27me. Genes with higher H3K27me3 enrichment in queen-right workers (magenta) (*A*) and queen-less workers (dark green) (*B*) are indicated by darker colored nodes. Interacting genes (queen-right workers, light magenta; queen-less workers, light green) were identified using BioGRID in DAVID. Network analysis was performed using Cytoscape. The predicted key hubs in this network have a high degree of centrality (as indicated by the relative size of each node) and are putative key regulators of reproductive constraint in honeybees.

Network analysis (fig. 5) of genes with differential enrichment of H3K27me3 identified the epidermal growth factor receptor (Egfr) as a key hub in repressed worker ovaries consistent with previously published work (Formesyn et al. 2014). Cyclin E was also identified as having a possible role in modulating ovarian repression, in *Drosophila* Cyclin E modulates responsiveness of germline stem cells to signals from the germline niche (Ables and Drummond-Barbosa 2013) and has been linked to PRC2 and H3K27me3 in this species (Iovino et al. 2013). In both queen-less and queen-right workers, Notch signaling (including gene encoding the ligands delta [Dl] and Serrate [Ser]) were identified as key hubs in the network consistent with our gene expression analyses (fig. 1*C*) and previous studies (Duncan et al. 2016) (fig. 5).

## Discussion

We provide empirical evidence that the honeybee genome is ordered into genomic regulatory domains with respect to ovarian plasticity. Thirty-five percent of the 2,912 genes that are differentially expressed between queen-right and queen-less honeybee ovaries lie in coregulated clusters of genes, many of which have been assembled during the evolution of the Apidae clade (fig. 2 and supplementary fig. 3, Supplementary Material online). The coregulated gene complexes we have identified are marked by H3K27me3, with enrichment for this mark in regions flanking the genomic regulatory domains. We suggest that these marked clusters have evolved as a result of a selective pressure for complexes of coexpressed genes to form to ease coordinated gene regulation, as seen in other eukaryotes (Boutanaev et al. 2002; Roy et al. 2002). This selective pressure would build complexes of coregulated genes as genome rearrangements occurred, leaving clusters of epigenetically coregulated genes in the genome. That all genes regulated by ovary activation are not in clusters may reflect that the selective pressure for these genes to be kept together or be brought together over evolutionary time may be small, or that not all genes are available to be moved in the genome, perhaps because they are coregulated with another set of genes involved in another process (supplementary fig. 2 and table 5, Supplementary Material online). The clusters we have identified may represent topologically associating domains (Szabo et al. 2019). Finding such clusters is unusual in insects where, apart from the Hox (Pace et al. 2016), runt (Duncan et al. 2008), and E(spl)-C (Duncan and Dearden 2010) complexes, evolutionary conserved clusters of coregulated genes have not been identified.

Clusters of genes that are more highly expressed in queen-right worker ovaries (“Queen responsive clusters”), containing genes involved in active repression of the ovary, have a longer evolutionary history than those associated with activation of the ovary (“plasticity responsive clusters”). This implies that ovarian repression in response to the presence of a queen is derived from genes or pathways involved with repression of the ovary due to diapause, seasonal variation, or nutritional deficiency. Our data imply that reproductive constraints in honeybees, key to the evolution of eusociality, evolved from extant and ancient systems for regulating the ovary in response to environmental stimuli.

In contrast, we show that clusters of genes associated with activation of the ovary (“plasticity responsive clusters”) are younger than those for repression of the ovary (“Queen responsive clusters”) and largely assembled “de novo” in bees. Little is known about how clusters of coregulated genes controlling polymorphic phenotypes are assembled over evolutionary time but recent simulations have indicated that phenotypic plasticity and fluctuating environments may result in the assembly of clusters of genes in the genome that are coregulated by the same plasticity modifier or transcription factor (Gulisija and Plotkin 2017). Our data, which show that plasticity responsive clusters are young and assembled “de novo” in bees (fig. 2), provide the first empirical support for this hypothesis. The assembly of these clusters into domains under the control of Notch signaling, a key plasticity modifier in this process (Duncan et al. 2016), may be a key part of the cooption of Notch signaling into control of ovary activation.

The clusters of plasticity responsive genes, and H3K27me3 marks around those clusters, imply that the honeybee genome is structurally and functionally organized to respond to the loss of the queen and her pheromone. Although honeybees exhibit extreme forms of plasticity, for example, ovarian plasticity and also the caste polyphenism, we observe that the genome of the honeybee is nonrandomly organized with respect to a range of gene expression responses, including these extreme forms of plasticity (supplementary fig. 2, Supplementary Material online). Strikingly, these gene expression responses are associated with largely different clusters of genes suggesting that selective pressures on different traits, including plasticity, have acted to shape honeybee genome organization. It seems likely that responses to environmental events may also shape the genomes of other eukaryotes. It will be important to examine the relationships between plasticity and genome organization in other species, especially humans, as this may give important insights into the architecture of plasticity-related illness. Response to environmental effects may have a crucial role in shaping the structure, organization, and regulation of animal genomes with consequences for our understanding of plasticity, genome evolution, and health.

## Materials and Methods

### Honeybee Husbandry and Ovary Tissue Collection

*Apis mellifera* were supplied by Betta Bees Research Limited, which maintain a closed breeding population of *A. mellifera* using instrumental insemination (Hyink et al. 2013). Bees were reared in Langstroth hives or nucleus boxes in Dunedin, New Zealand. Although all bees were obtained from this closed population, individuals were sampled from multiple hives across this population for this study as we wanted to ensure we had multiple genetic lineages present in our samples to increase the probability of identifying the biological phenomena underpinning phenotypic plasticity rather than interindividual or lineage specific events. Queen-right worker bees were obtained from honeybee colonies with a confirmed laying queen. Queen ovaries were taken from young, actively laying queens (∼12 months old). Queen-less honeybee colonies were established by removing frames containing brood and worker bees from queen-right hives into a nucleus box. Queen-less colonies were monitored for the presence of worker-laid eggs and cells with developing queens were destroyed.

Dissection of ovary tissue from queen-less worker bees was carried out at least 2–4 weeks after a queen-less hive was established. This is a sufficient period for all queen-laid brood to have emerged and generally, dependent on season (Velthuis 1970; Hoover et al. 2006), for worker-laid eggs to be detected. Ovary activity scores were based on a modified Hess scale (Hess 1942) as previously described (Duncan et al. 2016). Briefly, ovaries that were small, lacking defined ova and morphologically indistinguishable from queen-right worker ovaries were scored 0, ovaries that had thickened and had differentiated cells but no yolk deposition were scored 1, ovaries with developing oocytes and yolk deposition were scored 2, and ovaries with at least one mature ova were scored 3 (fig. 3*A*).

### RNA Extraction

Bees were cooled briefly at 4 °C to anesthetize them and then abdomens removed from the thorax. Ovaries were dissected from the abdomens in phosphate-buffered saline solution. Ovary activity was determined (score = 0–3) before snap freezing and storage at −80 °C. For each biological replicate, the number of individuals were queen (*n* = 3), queen-less worker ovary (score = 3) (*n* = 5), and queen-right worker ovaries (score = 0) (*n* = 40); sufficient tissue to yield >10 µg of total RNA. RNA extraction was carried out using Trizol reagent (Invitrogen) and purified and treated with DNAse using RNAeasy columns (Qiagen). Quantity of RNA was determined using spectrophotometry and quality via an Agilent Bioanalyzer.

### RNA-seq and Analysis

RNA-seq analysis was carried out using an Illumina HiSeq 2000 (Beijing Genomics Institute, BGI) on two independent biological replicates of queen, queen-right worker (score 0) and actively laying worker (score 3). RNA samples were quality controlled by gel electrophoresis and an Agilent Bioanalyzer, RNA integrity numbers were not used as a determinant of RNA quality as insect 28s rRNA denatures upon heating resulting in suboptimal RNA integrity number values (Winnebeck et al. 2010). Libraries were constructed and sequenced using standard methods by BGI using 10 µg of total RNA. Briefly, mRNA was isolated from total RNA using oligo(dT) magnetic beads, mRNA molecules were fragmented, and first strand cDNA synthesis performed using random hexamer primed reverse transcription followed by second-strand cDNA synthesis. The cDNA was subjected to end-repair and then was 3′ adenylated. Adapters were ligated to the 3′ ends, and the library was subjected to polymerase chain reaction (PCR) amplification and purified using AMPure XP beads (Agencourt). Libraries were validated on an Agilent 2100 bioanalyzer. Single end 50-bp reads were generated using an Illumina HiSeq 2000 by BGI. Adaptor trimming, removal of contamination, and low-quality reads were performed by BGI and verified by FastQC analysis (http://www.bioinformatics.babraham.ac.uk/projects/fastqc/; last accessed March 14, 2020). Read depth ranged from 6.96 to 7.5 million clean reads for each sample (supplementary table 4, Supplementary Material online). Read mapping, quantification, normalization, and differential expression analysis were carried out using CLC Genomics Workbench software version 7.2 (Qiagen). Reads were mapped to the *A. mellifera* genome (v4.5 from the NCBI FTP genome directory available at ftp://ftp.ncbi.nih.gov/genomes/Apis_mellifera; last accessed March 14, 2020), and gene expression levels determined by counting the number of reads that mapped to each gene model using the RNA-seq algorithm implemented in CLC Genomics, which is based on the approach of Mortazavi et al. (2008). The following parameters were used: Create fusion gene table = No, Create report = Yes, Create list of unmapped reads = Yes, Additional downstream bases = 500, Exon discovery = Yes, Minimum read count fusion gene table = 5, Minimum length of putative exons = 50, Minimum number of reads = 10, Maximum number of mismatches (short reads) = 2, Organism type = Eukaryote, Use annotations for gene and transcript identification = Yes, Expression level possible values: Genes, Transcripts = Genes, Use strand specific assembly = No, Unspecific match limit = 10, Additional upstream bases = 500, Use colorspace encoding = No, Minimum exon coverage fraction = 0.2, Minimum length fraction (long reads) = 0.9, Minimum similarity fraction (long reads) = 0.8, and Expression value = Read Per Kilobase of exon Model value. For each library 92.75–95.73% of reads mapped to the genome uniquely (supplementary table 4, Supplementary Material online). The distribution of transcription values for each sample was manually inspected using box plots, the distributions between samples were normalized using the quantile method (Bolstad et al. 2003), and data were transformed by adding a constant of 1 (to avoid issues with dividing by 0 when calculating fold-changes). RPKM (reads per kilobase [kb] per million mapped reads statistic [RPKM = total exon reads mapped/mapped reads in millions × exon length in kb]) for each annotated gene (11,158 genes) was calculated. Genes that were differentially expressed were identified using a Baggerly test (Baggerly et al. 2003) false discovery rate (FDR; *P* value < 0.01). RNA-seq data were validated using the expression of ten genes determined by reverse transcription quantitative PCR (RT-qPCR) (supplementary fig. 9, Supplementary Material online) using cDNA from independently generated RNA samples. These genes were selected as they ranged from relatively low expression (*GB10585*, *Notch* < 10 RPKM), to relatively high expression (*Vg*, *Yl*, *Aub* > 100 RPKM) and included genes that we had previously determined the expression of by RT-qPCR (Duncan et al. 2016).

*Drosophila* orthologs of the honeybee genes were obtained from the InParanoid database (Östlund et al. 2010). Differentially expressed genes with *Drosophila* orthologs were analyzed with the Database for Annotation, Visualization, and Integrated Discovery (DAVID) (Huang et al. 2009) and FlyMine (Lyne et al. 2007). Gene interaction networks were assessed using Cytoscape (Cline et al. 2007). All honeybee genes expressed in the ovaries that also had a *Drosophila* ortholog were used as the background list.

### Cluster Analysis

Physical clusters of differentially enriched genes were identified using CROC (Pignatelli et al. 2009) using the honeybee genome annotation file (gff3, v4.5) obtained from the NCBI FTP genome directory available at ftp://ftp.ncbi.nih.gov/genomes/Apis_mellifera. CROC uses the physical location (bp) of genes on chromosomes/contigs and determines whether genes within a list of interest, in this case differentially expressed genes, are localized within a cluster on the chromosome/contig. CROC analysis can be carried out on a gene-based (essentially ignoring physical distance between genes) or a DNA length-based/window analysis. We carried out CROC analysis on our data using both a gene-based and window-based approach (table 1). In both analyses, we used a minimum cluster size of three genes with a Benjamini and Hochberg corrected *P* value of <0.01. For window-based analysis, we used a window size of 50 kb and an offset window of 1 kb. CROC uses a hypergeometric distribution test to determine the probability of obtaining the number of genes (from the gene list of interest) in the current window by chance alone. This analysis was carried out independently on genes that were more highly expressed in queen-right workers (vs. queen-less workers), queen-less workers (vs. queen-right workers), queen-less workers (vs. queens), and queens (vs. queen-less workers) reflecting the biologically relevant comparisons that we undertook for our RNA-seq analysis (fig. 1*A*).

However, to determine whether the honeybee genome is structured with respect to plasticity, we also wanted to determine if we detected significantly more (or less) clusters within our gene lists than we would expect by chance. To do this, we took a bootstrapping resampling approach, where we performed 10,000 replicates of the CROC analysis, each replicate consisting of the same number of genes in our list of interest (differentially expressed genes) randomly sampled from our background list. The background list consisted of all of the genes that were expressed in the honeybee ovaries (i.e., had an RPKM of >5 in both biological replicates of at least queen, queen-right worker, or queen-less worker). We then calculated whether our lists of differentially expressed genes had more (or less) clusters than we would expect by chance based on our bootstrapping resampling analysis.

### Evolutionary History of Clusters

To determine the evolutionary history of these clusters across the hymenoptera, we identified orthologs and established order for genes within our gene clusters (table 1 and supplementary tables 1 and 2, Supplementary Material online) across 13 hymenopteran species. Peptide fasta files and gff3 (containing genomic features including genomic location for genes annotated on the genome) files were obtained for 14 species spanning the Hymenopteran phylogeny. We targeted seven-bee species (*Eufriesea mexicana v1.1*, *Bombus impatiens v1.0*, *Bombus terrestris v1.3*, *Dufourea novaeangliae v1.1*, *Melipona quadrifasciata v1.1*, *Habropoda laboriosa v1.2*, and *Megachile rotundata v1.1*) (Kapheim et al. 2015; Sadd et al. 2015) from The Hymenoptera Genome Database (http://hymenopteragenome.org/beebase/? q=consortium_datasets; last accessed March 14, 2020) (Elsik et al. 2016), four ant species (*Harpegnathos saltator* v3.3, *Pogonomyrmex barbatus* v1.2, *Atta cephalotes* v1.2, and *Acromyrmex echinatior* v 3.8) all from the Ant Genomes Portal (http://hymenopteragenome.org/ant_genomes/) (Bonasio et al. 2010; Nygaard et al. 2011; Smith et al. 2011; Suen et al. 2011), *Polistes canadensis* from NCBI (release 24/11/2015) (Standage et al. 2016), and *N. vitripennis* v2.1 (Werren et al. 2010) from Hymenoptera Genome Database (http://www.hymenopteragenome.org/?q=hymenopteramine_datasets) (Elsik et al. 2016). In each case, peptide fasta files and gff3 files were obtained. Orthologous and paralogous groups of genes were identified using a local installation of BLAST+ (v 2.7.1) (Camacho et al. 2009), and VESPA (v 1.0 Webb et al. 2017) and OrthoFinder (Emms and Kelly 2019). For genes where one-to-one orthology could not be clearly assigned, phylogenetic analysis using Bayesian techniques was undertaken. Briefly, protein sequences were aligned using Clustal Omega (v1.2.0) (Sievers et al. 2011) and Bayesian phylogeny constructed using MrBayes (v3.2.5) (Ronquist et al. 2012) with 1,000,000 generations, mixed models, and default priors. Samples were taken every 1,000 generations and the first 25% of runs were discarded as “burnin.”

Each orthologous gene family was manually inspected, and genomic coordinates for each gene extracted from the relevant gff3 files and clusters were visualized in their chromosomal location across the hymenopteran phylogeny (Peters et al. 2017) using genoPlotR (v0.8.9) in R (v 3.3). Heat maps were generated in R using ggplot2 and are based on the proportion of genes in the cluster where gene order is conserved among each species.

### Quantitative RT-PCR

Reverse transcription from RNA was carried out using VILO reagent (Invitrogen) to produce cDNA. cDNA (1:10 dilution) was used as a template for quantitative RT-PCR (RT-qPCR). Primer3plus (Untergasser et al. 2007) was used to design oligonucleotide primers that spanned exon/intron boundaries (supplementary table 6, Supplementary Material online), and these were evaluated using Beacon Designer (PREMIER Biosoft). The specificity of designed PCR primers was assessed using primer-BLAST (Ye et al. 2012). RT-qPCRs were carried out on a BioRad CFX Real-Time PCR detection system with SsoFast EvaGreen PCR mastermix, 5 ng of cDNA, and 300 nM of each primer. Three biological replicates were measured for each condition, and each measurement was made in duplicate. Gene expression analysis was carried out using the BioRad CFX (CFX Manager software version 3.1). Gene expression was normalized to the geometric mean (Vandesompele et al. 2002) of the relative quantities of two reference genes: *Rpn2* and *mRPL44* (Duncan et al. 2016). To determine if data fitted a normal distribution the Shapiro–Wilk test was used and differences in target gene expression were determined by ANOVA (Analysis of variance) with a Tukey’s post hoc test.

### Histone Extraction and Western Blot

The EpiQuik Total Histone Extraction Kit (Epigentek) was used to extract histones from honeybee ovary tissue. Qubit fluorometer and protein assay kits (Invitrogen) were used to estimate protein concentration. Ten micrograms of protein were separated on a 12% SDS PAGE gel at 200 V for 1 h in Tris-Glycine-SDS electrophoresis buffer and run alongside a molecular weight marker (Novex prestained ladder [Invitrogen]). Proteins were transferred to nitrocellulose membrane in Towbin’s buffer (25 mM Tris, 192 mM glycine, 0.1% SDS, pH 8.3, 20% methanol), the membrane was blocked in 2% bovine serum albumin in TBS-T before incubation with H3K27me3 antibody (Abcam 6002) (1:200) at 4 °C overnight. The membrane was washed and incubated with HRP-conjugate anti-mouse (Jackson Immunochemicals) (1:1,000) at room temperature for 1 h. The chemiluminescent reaction step was performed using the Pierce ECL Western Blotting substrate (Thermofisher). The blot was imaged using the Fuji LAS-3000 ECL imaging system. After detection of H3K27me3, the membrane was stripped for 10 min in stripping buffer (200 mM Glycine, 0.01% SDS, 0.1% Tween) and washed before incubation with the anti-Histone H3 Polyclonal Rabbit (abcam 1791) (1:1,000) and a second chemiluminescent reaction step.

### Drug Treatment of Honeybees

Drug treatment of honeybee workers was carried out as previously described (Duncan et al. 2016). Emerging brood were removed from hives and incubated at 35 °C overnight. Newly emerged bees were contained in 8 × 8 × 4 cm wooden cages (*n* = 100–120 bees per cage) at 35 °C. The cages had a section of empty comb attached to the rear wall of the cage. Bees were fed high-protein pollen cake (replaced daily) and water was given ad libitum. DZnep (an inhibitor of E(z)) (Tan et al. 2007) (Abcam ab145628) was dissolved in water and mixed into the food at a final concentration of 50 μM. Food intake and lethality were recorded (supplementary fig. 6, Supplementary Material online) daily. Experimental treatments were carried out in triplicate on two independent occasions. After 10 days, bees were euthanized and their ovaries were dissected and photographed using a Leica Mz75 stereomicroscope with a DFC280 digital camera and Leica Application Suite software (v. 2.5.0.R1). Randomized photographs were scored blindly by two people, using the scale described (fig. 3*A*). Differences between control and treated cages were determined using a Fisher’s exact test for proportions of each ovary-activation class between treatments.

### Chromatin Immunoprecipitation

Freshly dissected ovary tissue was homogenized using a 22-gauge hypodermic needle and syringe in phosphate-buffered saline and then transferred to a dounce homogenizer for 20 strokes of the B pestle. Chromatin extraction, shearing, and immunoprecipitation were performed using the Magnify ChIP Kit (Invitrogen) and a ChIP grade antibody for H3K27me3 (Abcam ab6002). Chromatin was sheared to ∼200 bp using a Covaris AFA and 10 μg of chromatin was used for each ChIP reaction. Biological duplicates were generated for queen ovary, queen-less worker ovary, and queen-right worker ovary tissue. DNA from technical triplicate ChIP reactions was pooled for each biological replicate sample and then air dried in a vacuum centrifuge before resuspending in Elution Buffer. The size and concentration of the immunoprecipitated DNA was determined with an Agilent 2100 Bioanalyzer and a high sensitivity DNA kit. ChIP DNA (10 ng) was sequenced on an Illumina HiSeq 2000 (Beijing Genomics Institute). ChIP-qPCR was carried out to validate the ChIP-seq data (supplementary fig. 10, Supplementary Material online). H3K27me3 immunoprecipitated samples were normalized to percentage input after correction for primer amplification efficiency.

### Analysis of Chromatin Immunoprecipitation

Honeybee genome sequences (v4.5) in fasta format were obtained with the corresponding gff3 file from the NCBI FTP genome directory available at ftp://ftp.ncbi.nih.gov/genomes/Apis_mellifera. Bowtie (Langmead et al. 2009) was used to map the raw sequencing reads in fastq format to the honeybee reference genome version 4.5 using default parameters. SAM (sequence alignment/map) format was converted to BAM (binary alignment/map) format using samtools (Li et al. 2009).

### Identifying Genomic Regions Differentially Enriched for H3K27me3

Two approaches were taken to identifying differentially enriched areas of the honeybee genome: MACS2 (v2.1.1.20160309) (Zhang et al. 2008) was used to call peaks of H3K27me3 enrichment and DiffReps (Shen et al. 2013) was used to identify enriched regions using a sliding window approach, an approach that has been successfully used to analyze ChIP-seq data in honeybees (Wojciechowski et al. 2018).

MACS2 peak calling was used to identify regions of the genome where H3K27me3 was significantly enriched in the treatment group compared with the input control. MACS2 was run using broad peak calling with an *extension size* of 147. Unique peaks were identified using Bedtools intersect, and differential peaks were identified using ChIPcomp (Chen et al. 2015), which allows differential peaks to be identified taking into account the biological replicates.

Peak calling for histone marks can be difficult due to the broad and diffuse nature of the peaks (Pauler et al. 2009), therefore we used DiffReps (Shen et al. 2013), which uses a sliding window approach to scan the genome and identify windows with differential enrichment. *BED* files were analyzed using an exact negative binomial test (Anders and Huber 2010), the window size was set to 200 bp with a slide of 20 bp. Normalization of read counts within 200-bp windows was performed in diffReps. Only windows that pass an initial uncorrected *P* value (*P < *1e^−04^) cutoff were retained. Windows that passed this cutoff were adjusted for multiple comparisons using a FDR (Benjamini and Hochberg 1995).

Sequencing reads were assigned to genomic features of interest including genes and the TSS of genes after mapping to the reference genome using the featureCounts function (Liao et al. 2014) in the bioconductor (Gentleman et al. 2004) package Rsubread. Statistical analysis of the feature counts was performed using the bioconductor limma package (Smyth 2005). Genomic features associated with the regions of interest were identified using ChIPseeker (Yu et al. 2015). Differences in the distribution of these peaks across these genomic features were assessed using a Chi-squared distribution. *Drosophila* orthologs of genes associated with peaks or areas of enrichment were identified and proteins interacting with these differentially enriched genes were identified using DAVID (https://david.ncifcrf.gov/home.jsp) (Huang et al. 2009) as we hypothesized that these interactions may represent key hubs in a network linking together our differentially enriched genes. These interaction networks were visualized in Cytoscape (v3.6.1).

### Patterns of Enrichment around Clusters

In order to determine if H3K27me3 showed different patterns of enrichment within gene clusters, as opposed to flanking regions, bdgcmp within MACS2 (v2.1.1.20160309) (Zhang et al. 2008) was used to calculate fold enrichment of ChIP samples relative to input (background) across the whole genome for our queen-right, queen-less, and queen ovary samples. The grep command was used within the Linux environment to extract relevant the fold enrichment for H3K27me3 from the genomic regions encoding each of the gene clusters identified (table 1). Because these clusters vary in length (both in terms of gene content and absolute sequence length, supplementary fig. 1, Supplementary Material online), we expressed H3K27me3 enrichment relative to the length of the cluster; where 0 is the beginning of the cluster and 1 marks the end of the cluster. We also wanted to compare the patterns of H3K27me3 enrichment across the cluster with the flanking regions. To do this, we calculated H3K27me3 fold enrichment at half the length of the cluster both up and down stream of the gene cluster (e.g., if cluster spanned 10 kb, each flank was 5 kb expressed on the graph as −0.5 to indicate half the length of the cluster upstream and +1.5 to indicate half the length of the cluster downstream). To test whether the levels of H3K27me3 enrichment vary across these gene clusters, we calculated mean fold enrichment across the 5′ flanking regions, gene cluster, and 3′ flanking regions (fig. 4*B* and *D*), and differences in H3K37me3 enrichment in these regions were evaluated using Wilcoxon rank sum test with the null hypothesis that there would be no difference in enrichment on the flanks of the cluster versus the cluster body as we see when we examine individual genes within the clusters (supplementary fig. 7, Supplementary Material online).

### Immunohistochemistry

Immunohistochemistry was used to determine cell type specificity of the H3K27me3 mark and was carried out as previously described (Dearden et al. 2009; Duncan et al. 2016) using H3K27me3 antibody (Abcam ab6002) (1:100) and AlexaFluor 637 antibody (Invitrogen) (1:250). 4',6-diamidino-2-phenylindole was used as a counterstain for nuclei (supplementary fig. 11, Supplementary Material online).

### Code Availability

The R script that was written to take 10,000 random subsamples of a background gene list to determine if clusters of genes occur significantly more (or less) often in a gene list of interest than they do in a background gene list is freely available at https://github.com/ejduncan/ClusterAnalysis

### Data Availability

Data underpinning this manuscript have been submitted to the Gene Expression Omnibus (GEO), reference number GSE120563. (https://www.ncbi.nlm.nih.gov/geo/query/acc.cgi?acc=GSE120563).

## Supplementary Material

Supplementary data are available at *Molecular Biology and Evolution* online.

## Author Contributions

E.J.D. performed and analyzed RNA-seq experiments and cluster analysis, contributed to the analysis of the ChIP-seq data, prepared figures, and cowrote and edited the final manuscript. M.P.L. did drug trials, immunohistochemistry, RT-qPCR PRC2 genes, and ChIP; contributed to the analysis of the ChIP-seq data; contributed to figure preparation; and cowrote the manuscript. P.K.D. proposed and planned the experiments, contributed to data analysis, contributed to figure preparation, and cowrote and edited the final manuscript. All authors read and approved the final manuscript.

## Supplementary Material

msaa057_Supplementary_Data
