# Supplementary material for: Genome Architecture Facilitates Phenotypic Plasticity in the Honeybee (Apis mellifera)
Source: Mol Biol Evol. 2020 Apr 5;37(7):1964–78. doi: 10.1093/molbev/msaa057 (PMC7306700; doi:10.1093/molbev/msaa057)
Supplement: msaa057_Supplementary_Data [file msaa057_supplementary_data.pdf]

Supplementary Materials for

Genome architecture facilitates phenotypic plasticity in the honeybee (*Apis mellifera*).

Elizabeth J. Duncan[1,2*], Megan P. Leask[1*], Peter K Dearden[1§].

1 Genomics Aotearoa and Biochemistry Department.
University of Otago
Dunedin
Aotearoa-New Zealand

2 School of Biology,
Faculty of Biological Sciences,
University of Leeds,
Leeds LS2 9JT
UK

*These authors contributed equally to this work
[§]To whom correspondence should be addressed.

This PDF file includes:

Supplementary Text

Supplementary Figures S1 to S11

1. Properties of plasticity associated gene clusters.
2. Gene clusters in common between published honeybee gene expression datasets.
3. Graph summarising the evolutionary history plasticity-related clusters
4. Summary of the evolutionary history of an example plasticity-related cluster.
5. Full western blot
6. Food intake and Kaplan-Meier survival curves for DZNep treated bees.
7. Patterns of enrichment of H3K27me3 amongst individual genes within the clusters.
8. Genome wide patterns of H3K27me3 in honeybee ovaries
9. Validation of RNA-seq by RT-qPCR.
10. ChIP-qPCR validation of ChIP-seq data.
11. Cell type enrichment of H3K27me3 in honeybee ovaries

Supplementary Tables S1 to S6

1. Clusters of genes more highly expressed in queen-right worker ovaries as compared with queen-less worker ovaries
2. Clusters of genes more highly expressed in queen-less worker ovaries as compared with queen-right worker ovaries
3. Clusters of genes more highly expressed in queen-less worker ovaries (as compared with queen ovaries
4. Sequencing and mapping statistics for RNA-seq and ChIP-seq data.
5. Clusters of genes associated with differential expression in previously published A. mellifera RNA-seq datasets
6. Oligonucleotide primer sequences used in this study

Supplementary text

Clusters of genes found when comparing gene-expression in queen ovaries and queen-less worker ovaries.

In this comparison very few genes were differentially expressed (Figure 1A). However, amongst the 27 genes that were more highly expressed in queen-less worker bee ovaries (as compared with queen) a single cluster of genes was detected. This cluster consists of three closely linked genes (Supplementary Table 3) spans 15,882 bp of the genome. These sequences are highly similar at the sequence level indicating they have arisen as a result of gene duplication. Phylogenetic analyses indicates recurrent duplication of these genes across the hymenoptera, with little sequence divergence. The phylogenetic relationships between these sequences don't recapitulate the species tree consistent with recurrent and gene conversion. We don't therefore include the analysis of this cluster in any further analyses.

Genome features associated with differential enrichment of H3K27me3

Mapping peaks of H3K27me3 enrichment to gene features across the honeybee genome in worker, active worker and queen ovaries indicates that this mark is most commonly found in intronic and distal intergenic regions, and that this global enrichment pattern is consistent between the three ovary states (Supplementary Figure 8A). Peaks of H3K27me3 are detected just 3' of transcriptional start sites of genes in queen, queen-less worker and queen-right worker ovaries (Supplementary Figure 8B), consistent with analyses of H3K27me3 enrichment in ants (Simola, et al. 2013).

Relative to background, we see more differential peaks than expected enriched in promoter regions of genes in both queen-right and queen-less worker ovaries (Supplementary Figure 8C).

As H3K27me3 marks often appear in broad peaks that may be difficult to detect using peak calling software (Pauler, et al. 2009), we also used a sliding window approach (Shen, et al. 2013) to identify regions of differential enrichment (Supplementary Figure 8D). Differentially enriched peaks (Supplementary Figure 8C) were associated with different genomic features than differentially enriched windows (Supplementary Figure 8D) which may reflect the fact that sharper regions of enrichment, more likely to be called as peaks, are associated with particular genomic features, including promoter regions (Supplementary Figure 8A).

Comparing genomic windows enriched for H3K27me3 revealed a complex pattern of dynamic changes in the transition from queen-right to queen-less worker ovaries (Supplementary Figure 8D). Queen-right workers have an over-representation of H3K27me3 enrichment in exons of genes and first introns relative to background and queen-less workers, which may reflect H3K27me3 mediated repression of gene expression (Simola, et al. 2016). Both queen-right and queen-less workers also have more H3K27me3 enrichment in distal intergenic regions than expected which may represent enhancer regions or chromatin domains (Evans, et al. 2016).

Despite observing very few differences in gene expression when comparing queen-less workers to queens (Fig 1A), we do see a number of differences in the H3K27me3 peaks in these tissues, however these differentially enriched peaks are not associated with any particular genomic feature (Supplementary Figure 8E).

Comparing genomic windows differentially enriched for H3K27me3 between queen-less and queen-right tissues revealed a complex pattern of dynamic changes (Supplementary Figure 8F), with a similar numbers of differentially enriched windows identified for this comparison as for the comparison of queen-right and queen-less worker ovaries (Supplementary Figure 8D), indicating differences in the H3K27me3 epigenetic landscape that is not reflected in gene expression (Figure 1A). In particular we see more highly enriched windows in queens associated with exonic regions, and in queen-less workers in intronic and distal intragenic regions, but the biological significance of these differences remains to be determined.

Network analysis of genes with differential enrichment of H3K27me3 in queen-less workers (Supplementary Figure 8G) and queens (Supplementary Figure 8H) didn't identify any key hubs in these regulatory networks unlike the analysis performed for queen-right and queen-less workers (Figure 5). This may indicate that differences in the epigenetic landscape seen between queen and queen-less worker tissue are independent of gene expression (consistent with our RNA-seq data, Figure 1) and may reflect a role unrelated to gene expression for H3K27me3 in these tissues.

Localisation of H3K27me3 within ovarian cell types

Honeybee ovaries are made up of different cell types (Supplementary Figure 11A), so we examined localisation of H3K27me3 using immunofluorescence to identify cell-type specific shifts in H3K27me3 (Supplementary Figure 11B).

In all three tissue types; queen, queen-right worker and queen-less worker ovaries, H3K27me3 is present at high levels in the cells of the terminal filament adjacent to the region where oocytes are specified (left panels Supplementary Figure 11B).

H3K27me3 is also present at high levels in the oocyte nucleus in early-oogenesis in queen and queen-less ovary tissue (stage 1, Supplementary Figure 11B, (Wilson, et al. 2011)); likely reflecting a role in transcriptional silencing of the oocyte (Iovino, et al. 2013; Prokopuk, et al. 2017).

H3K27me3 is also enriched in nuclei of terminal filament cells of repressed worker ovaries, however high levels of H3K27me3 are detected in nuclei of cells within the germarium where oocyte specification would be expected to take place. In the vitellarium of repressed worker ovaries, multiple nurse cells surrounding the arrested oocytes contain high levels of H3K27me3 and H3K27me3 in the oocyte nuclei is reduced, which may indicate misspecification of cell types in late oogenesis (Bottom Right Panel, Supplementary Figure 11B).

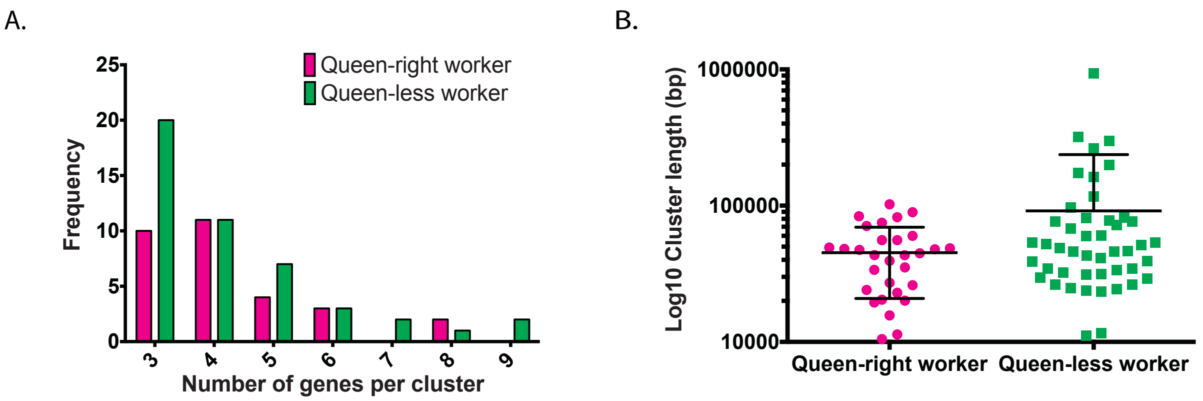

Figure S1 – Properties of plasticity associated gene clusters.

There was no statistically significant difference between the number of genes in a cluster (A) or the cluster lengths (B) between clusters in which expression is higher in queen-right worker (magenta) ovaries compared to clusters in which expression is higher in queen-less worker (green) ovaries.

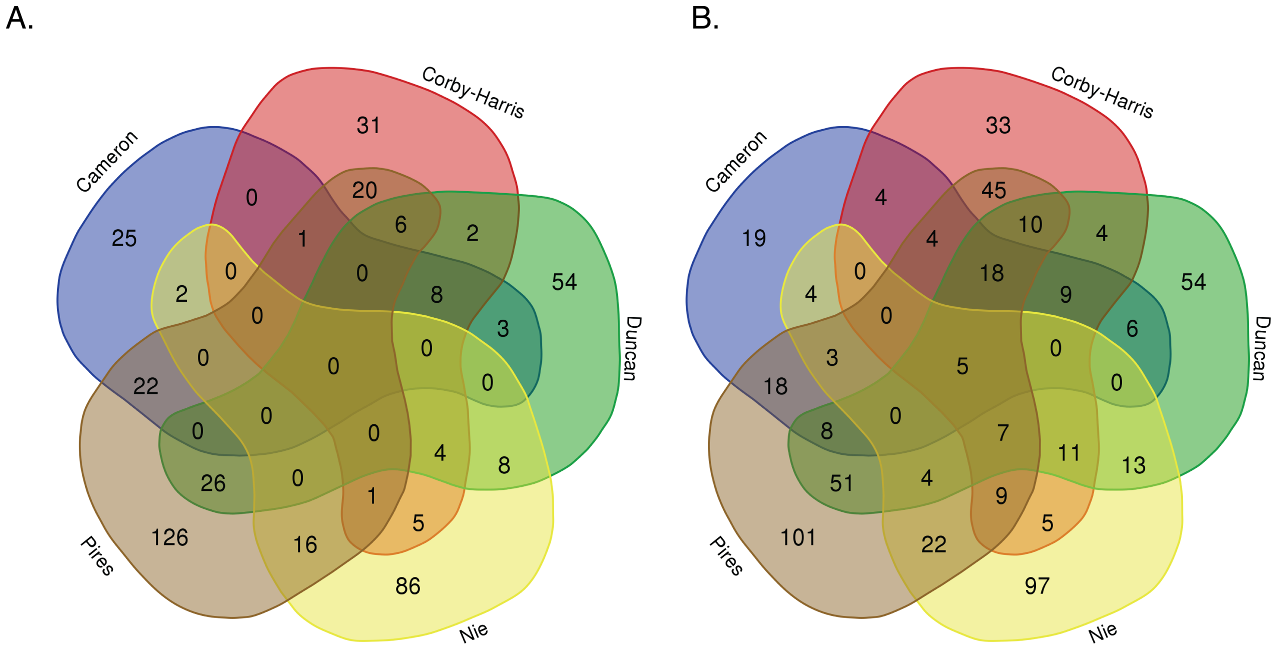

Figure S2 – **Gene clusters in common between published honeybee gene expression datasets.**

Venn Diagram showing overlap of gene clusters found in this study with those identified in previously published *A. mellifera* datasets (Cameron, et al. 2013; Corby-Harris, et al. 2014; Pires, et al. 2016; Nie, et al. 2018) using A) gene-based analysis, or B) window-based analysis in CROC (Pignatelli, et al. 2009). Full details of this analysis (including statistical analysis of whether clusters are over- or under-represented in these datasets) is provided in Supplementary Table 5.

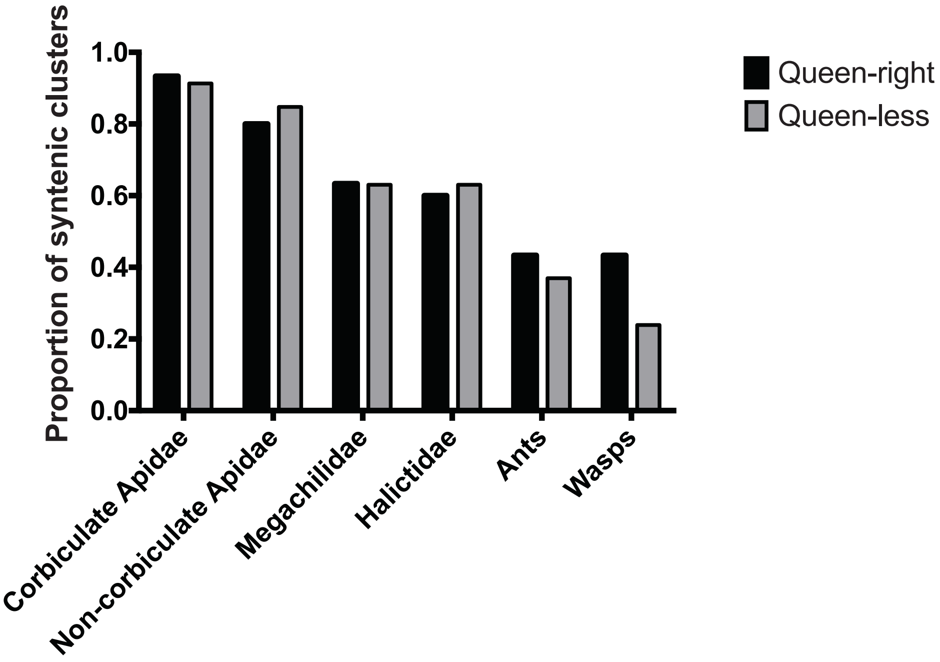

Figure S3 – Graph summarising the evolutionary history plasticity-related clusters
Bar graph showing the proportion of gene clusters identified in our honeybee RNA-seq data set (Table 1, Figure 2) that are conserved at the level of 75% conservation of gene order in other hymenopteran groups. Clusters of genes that are more highly expressed in queen-right worker ovaries 'QMP responsive clusters' (dark bars) have a similar evolutionary history as compared with clusters that are more highly expressed in queen-less worker ovaries 'Plasticity responsive clusters', except that more of the queen-right worker clusters are conserved at greater evolutionary distances, in particular in wasp species (divergence time ~140 mya (Misof, et al. 2014)) .

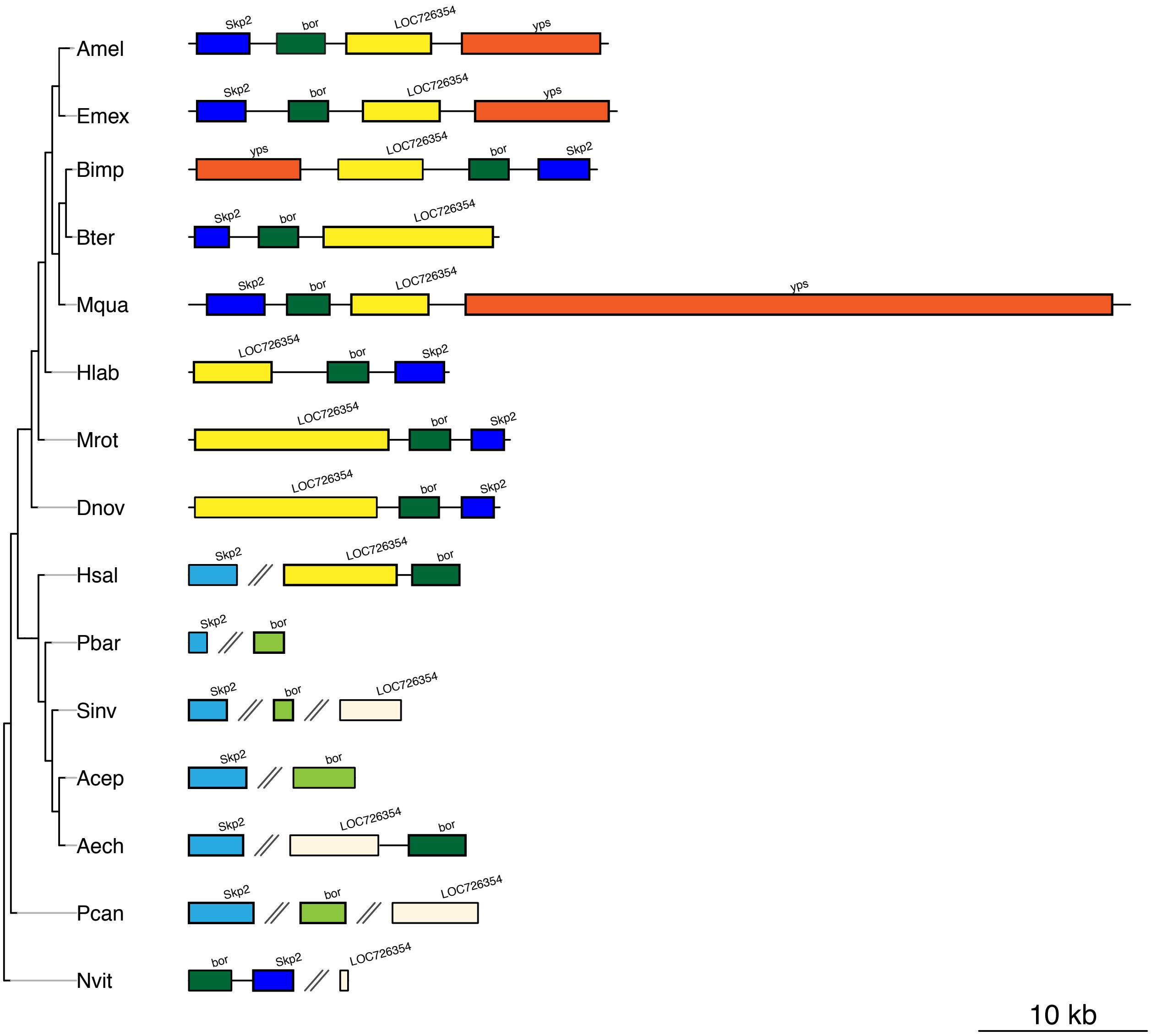

Figure S4 – Summary of the evolutionary history of an example plasticity-related cluster. In honeybees this gene cluster consists of four genes (Skp2, bor, LOC726354 and yps) that are located on LG16 (NW_003377872.1) with the cluster spanning 23.3 Kb. The figure shows a stylised phylogeny of hymenopteran species used in this study to examine the evolution of the gene clusters by determining the conservation of gene order. Orthogs are indicated by colour Skp2 (blue), Bor (green), LOC726354 (yellow) and yps (orange). When genes are joined by a solid line, this indicates that these genes are linked in that species and the use of a breakpoint indicates that the orthologs are present on a diifferent chromosome or contig indicating that gene order is not conserved. Scale (10 Kb) is shown at bottom right to give an indication of the relative sizes of the genes (intron and exon sequences) and cluster in each species.

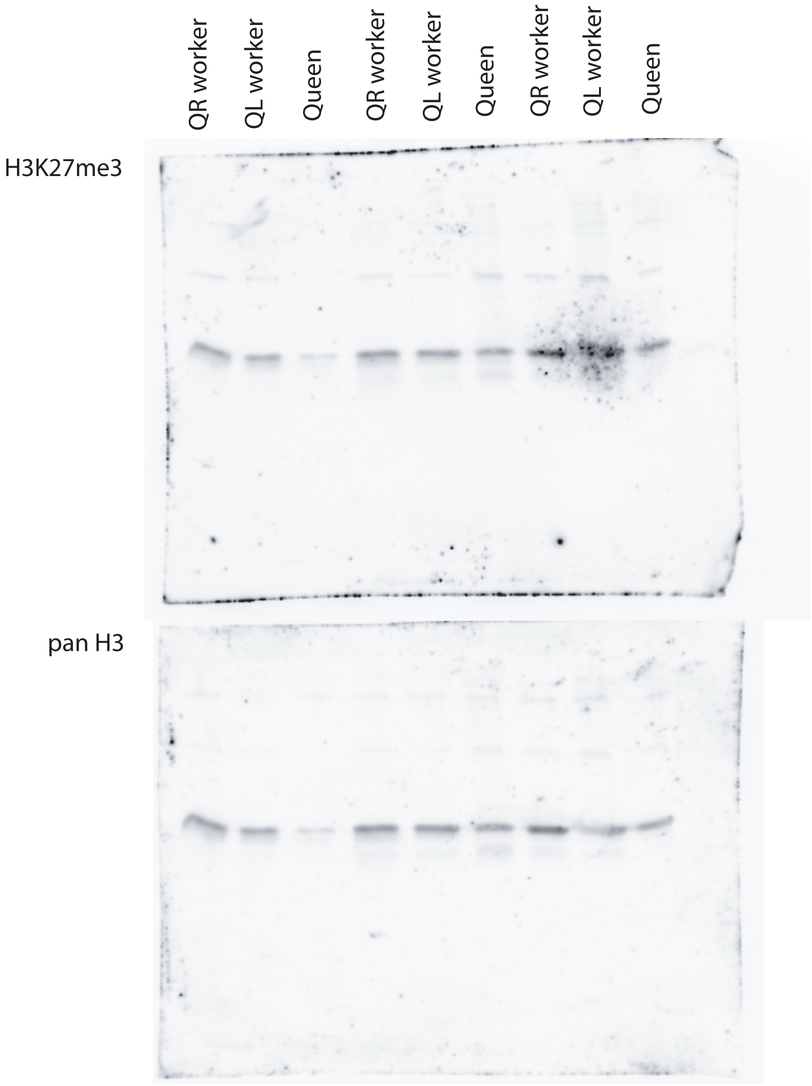

Figure S5 – Full western blot

Figure shows full Western blot analysis of ovary histone extracts for enrichment of H3K27me3 in queen, queen-right worker and queen-less worker ovary (as shown in Figure 3). The membrane was stripped and re-probed with Pan H3 antibody as the loading control. Western shows 5 μg of histone extract from queen-right worker, queen-less worker (score 3) and queen and was carried out in triplicate. H3K27me3 was used at 1:200 and anti-mouse-HRP secondary antibody was used at 1:1000.

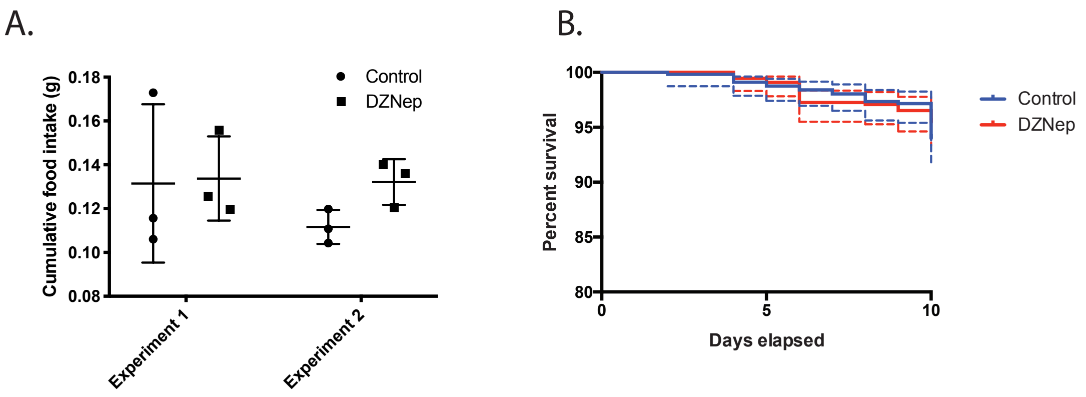

Figure S6 – Food intake and Kaplan-Meier survival curves for DZNep treated bees. In the absence of QMP, supplementation of the diet with DZNep had no significant effect on food intake (A, circles = solvent control (water), squares = DZNep treated) or survival (B) as compared with the solvent control. Dotted lines on Kaplan-meier survival curves are 95% confidence intervals. Statistical significance for food intake was measured using t-tests with a Holm-Sidak correction for multiple testing and for survival was measured using a log rank test.

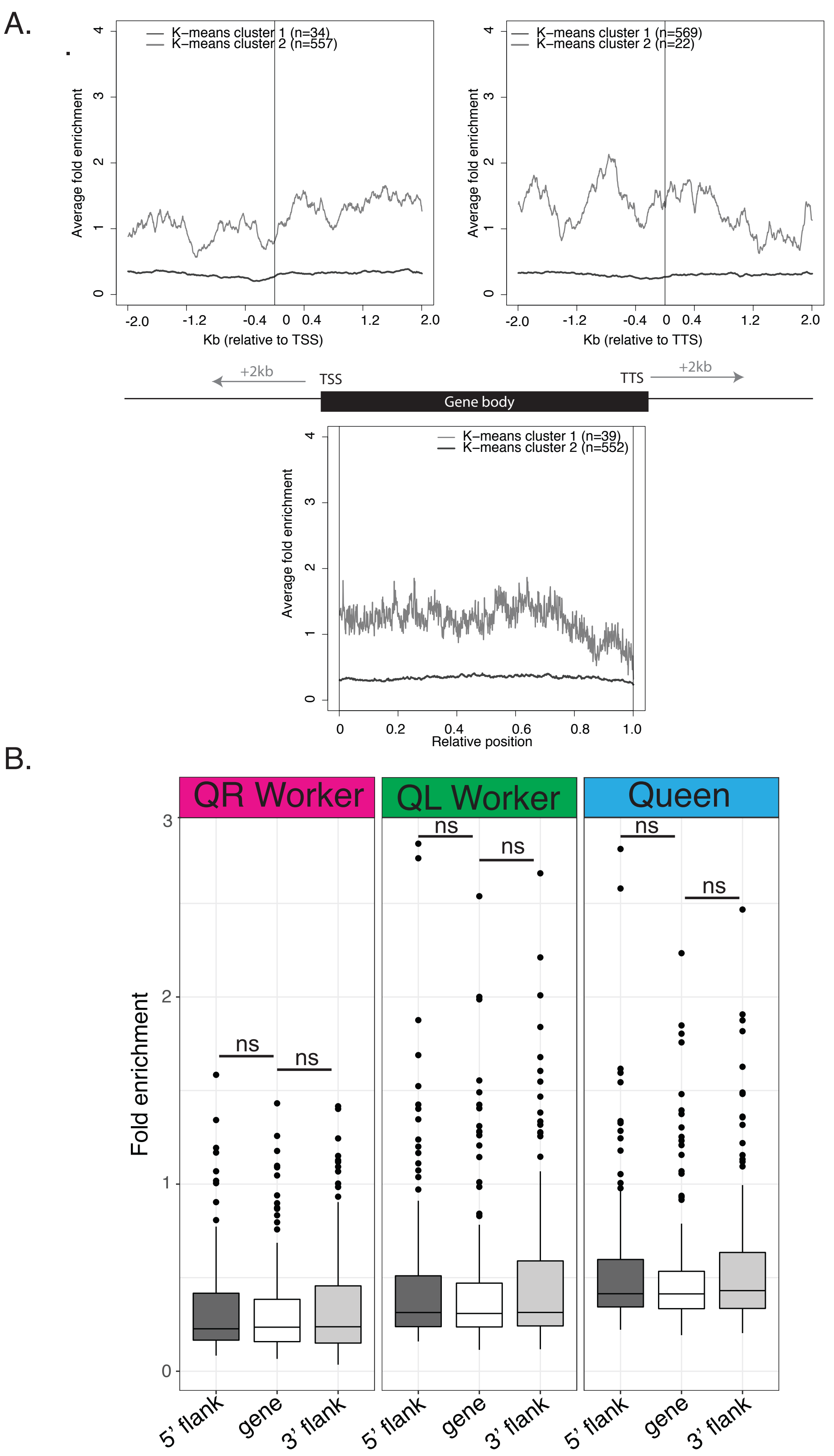

Figure S7 – Patterns of enrichment of H3K27me3 amongst individual genes within the clusters. To determine if the pattern observed in Figure 4 is an artefact associated with the way that individual genes are marked with H3K27me3, we examined the pattern of H3K27me3 within genes associated with gene clusters.

A) We first examined the H3K27me3 enrichment flanking the transcriptional start site (TSS), and transcriptional termination site (TTS) as well as across the gene body. The enrichment of H3K27me3 was found to show two patterns across these genes, with the majority of genes (n indicated in diagram) showing little change in H3K27me3 enrichment across the gene body, TTS or TSS. Some genes were however more dynamic in their H3K27me3 enrichment showing peaks of enrichment near the TSS and proximal to the TTS and a general decrease in enrichment towards the 3' end of the gene body. B) We also examined whether individual genes showed higher enrichment for H3K27me3 in the flanking regions as opposed to the gene bodies (as seen for whole clusters Figure 4) for individual genes contained within the cluster. No significant differences (ns) in enrichment were found. We conclude that no pattern of enrichment observed within the individual genes can explain the patterns of H3K27me3 we see associated with the gene clusters (Figure 4) indicating that the observed pattern is not an artefact reflecting the way that individual genes are marked but a real biological phenomenon.

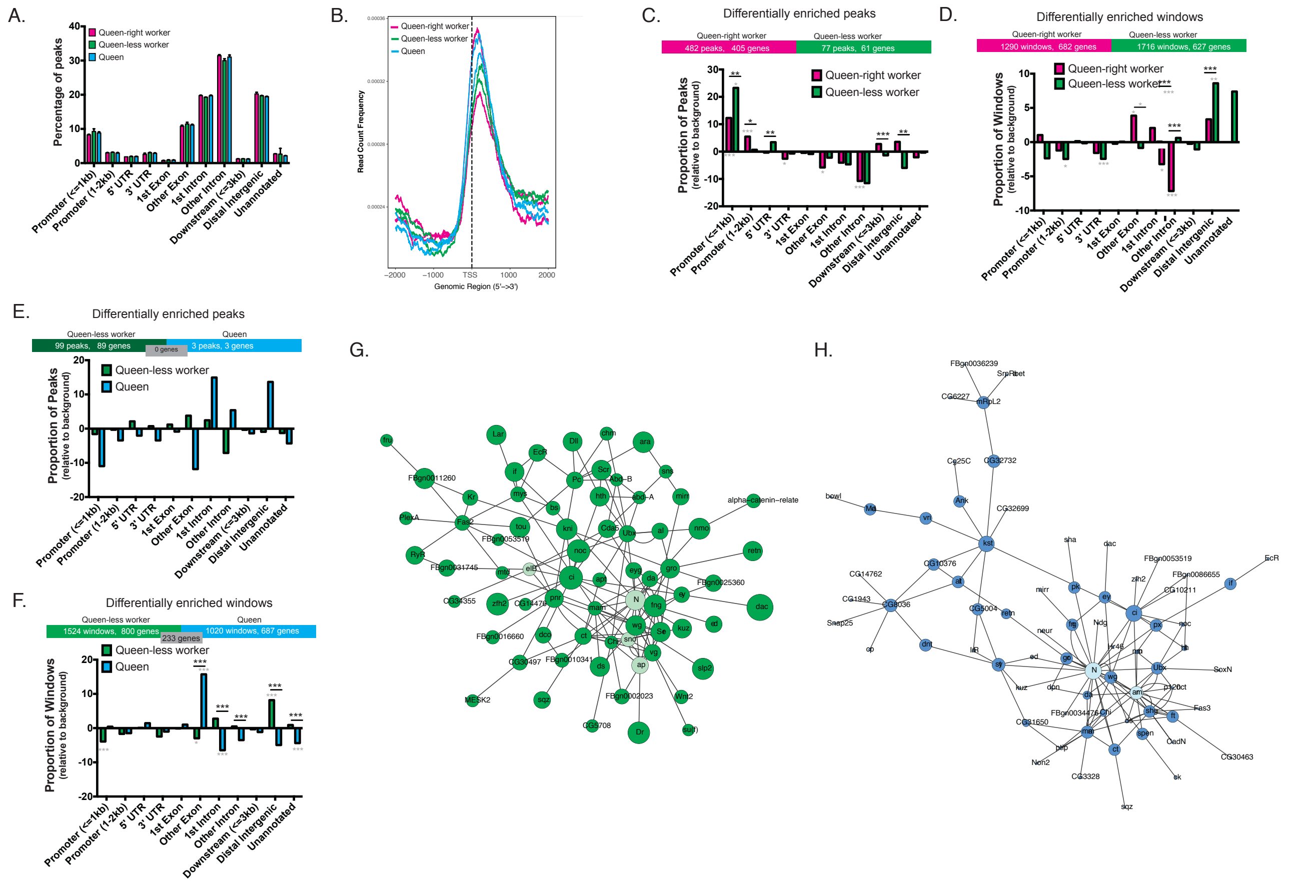

Figure S8. Genome wide patterns of H3K27me3 in honeybee ovaries. A) Across the genome the majority of H3K27me3 peaks are associated intronic and distal intergenic regions and there is no global shift in the genomic features that are associated with peaks between queen-right, queen-less and queen ovaries. B) Consistent with published data in other species H3K27me3 is enriched in a peak in all samples just distal to the transcriptional start site. C) Although we don't see a shift in global patterns of H3K27me3 with respect to queen-right and queen-less worker ovaries, when we examine individual peaks that are differentially enriched between these conditions we see that they are associated with different genomic features compared with background (significance indicated by grey asterisks) and also between queen-right and queen-less conditions (significance indicated by black asterisks). In particular, differentially enriched peaks are more associated with the promoter regions than we would expect (* p<0.05, ** p<0.01, ***p<0.001). D) D) Similarly, we also see differences in H3K27me3 enrichment in genomic windows between queen-right and queen-less workers. Differentially enriched windows are more over-represented in exons in repressed workers and this shifts to over-representation in distal intergenic regions in activated-workers. E) Despite observing very few changes in gene expression (Figure 1) when comparing queen-less workers to queens, we do see a number of differences in the H3K27me3 peaks in these tissues, however these differentially enriched peaks are not associated with any particular genomic feature. F) We also see a substantial number of differentially enriched windows of H3K27me3 when comparing queen-less worker and queen ovarian tissue, indicating differences in the H3K27me3 epigenetic landscape that is not reflected in gene expression. In particular we see more peaks in queens associated with exonic regions, and in queen-less workers distal intragenic regions, but the biological significance of these differences remains to be determined. Significance indicated by asterisks, * p<0.05, ** p<0.01, ***p<0.001. Genes with higher levels of H3K27me3 enrichment in queen-less workers (green, G) and queens (blue, H) are indicated by darker coloured nodes. Interacting

genes (queen-less workers = light green and queen = light blue) were identified using the BioGRID database via DAVID (Huang, et al. 2009). Network analysis was performed using Cytoscape (Cline, et al. 2007). Note that in both networks Notch was identified as a key regulator, but based on centrality analysis there were no key nodes identified in these networks unlike those identified when comparing queen-right to queen-less workers (Figure 5). This may indicate that differences in the epigenetic landscape seen between queen and queen-less worker tissue are independent of gene expression (consistent with our RNA-seq data, Figure 1) and may reflect a role unrelated to gene expression for H3K27me3 in these tissues.

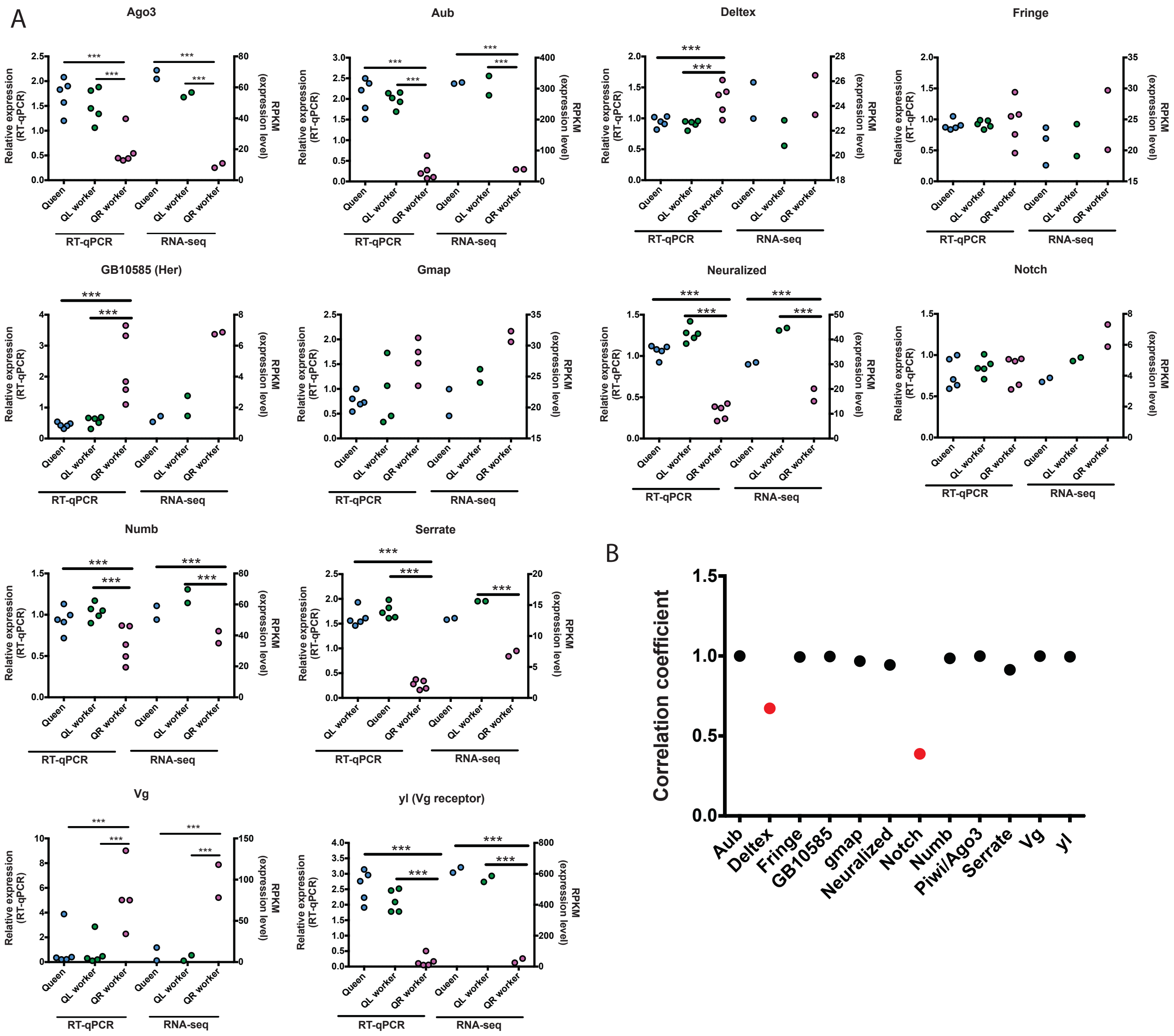

Figure S9 – Validation of RNA-seq by RT-qPCR.

Grpahs comparing the relative expression of selected genes determined by RT-qPCR (left hand set of three samples) compared with RPKM determined from RNA-seq data (right hand set of three samples). In each graph, data from queen ovaries is shown in cyan, queen-less workers in green and queen-right workers in magenta. RT–qPCR data is the mean of five biological samples for each condition, RPKM is the mean of two biological replicates per condition. Differences in gene expression in RT-qPCR data were determined by analysis of variance with a Tukey's post hoc test, and differences in gene expression in the RNA-seq data were determined using a Baggerly test with an FDR correction for multiple testing. Statistical significance is indicated above the bars (* p<0.05, ** p<0.01, ***p<0.001) B) Graph of the Pearson correlation coefficients generated by comparing RT-qPCR and RNA-seq data. Of the 12 genes tested in this study 10 are highly correlated with respect to RT-qPCR and RNA-seq data, with only *deltex* and *Notch* showing different patterns of expression in the RNA-seq and RT-qPCR data.

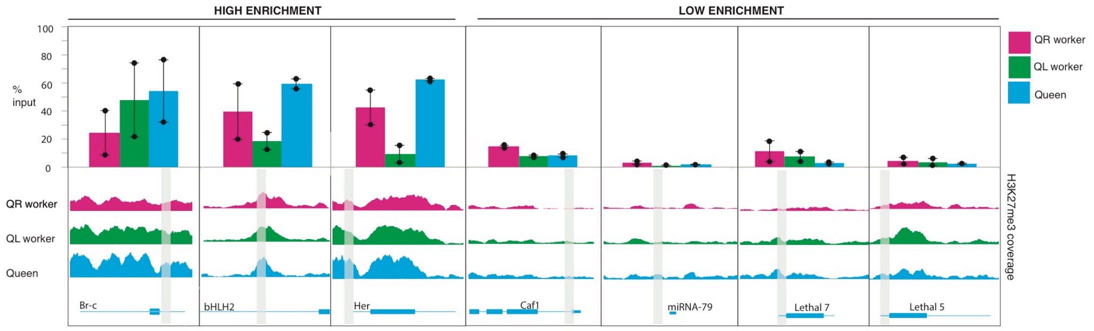

Figure S10 – ChIP-qPCR validation of ChIP-seq data. Three regions of high H3K37me3 and four regions of low H3K27me3 enrichment were selected for validation by ChIP-qPCR. A) ChIP-qPCR validation is shown in the bar graphs and error bars are the standard deviation of two biological replicates. B) ChIP-seq data for the relevant regions is shown below the ChIP-qPCR data and the y-axis indicates fold-enrichment relative to input (max = 4.0, sample size n = 2). C) Gene models are shown with the regions targeted by qPCR indicated as grey boxes and primer sequences in Table S6.

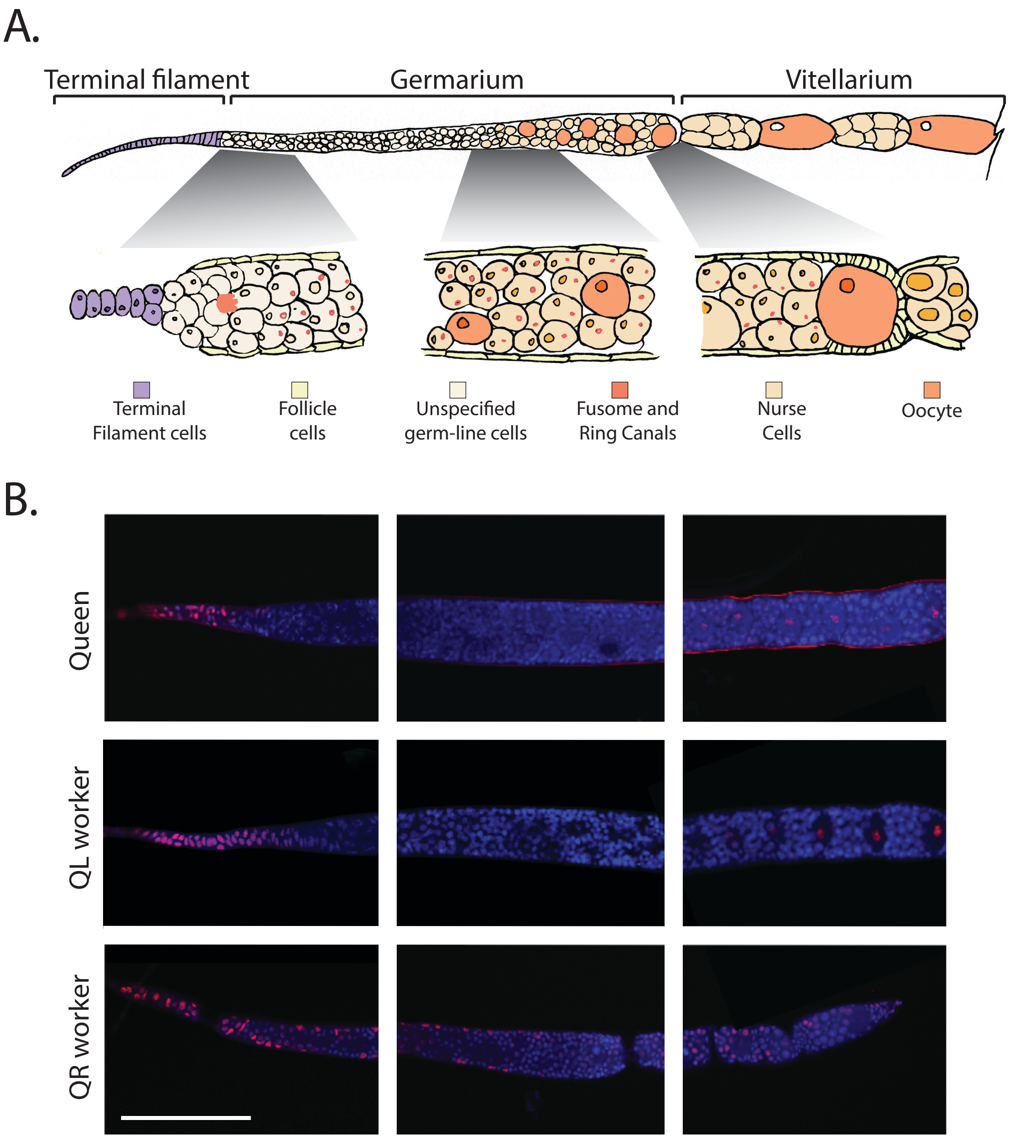

Figure S11 – Cell type enrichment of H3K27me3 in honeybee ovaries A) Schematic of the honeybee ovary illustrating general features of cell organization and the early stages of oogenesis in honeybee ovaries. B) Immunohistochemistry for H3K27me3 in honeybee ovaries. The anterior of the ovaries is to the left and the posterior is to the right. Scale bars represent 100 μm.

Supplementary Table 1: Clusters of genes more highly expressed in queen-right worker ovaries as compared with queen-less worker ovaries.

Contig	Start	End	Genes
**NW_003377909.1**	131693	142178	Apd-2 Apd-1 Apd-3
**NW_003377938.1**	1340295	1360388	LOC408559 LOC408557 Metap2 Ndufb2
**NW_003377939.1**	142302	176114	LOC726297 Crc LOC727647 Echs1
**NW_003377965.1**	21550	41017	LOC724405 LOC724488 LOC100576174 LOC410087 LOC724449 LOC724274 LOC724231 LOC724367
**NW_003377972.1**	882860	942841	Ppt1 LOC409495 LOC412589 LOC412416 LOC727483
**NW_003377974.1**	294779	334123	SP10 LOC724208 LOC409378 LOC411720
**NW_003377989.1**	1220196	1263459	LOC551946 LOC551968 Dhpr LOC552018 mRpL43
**NW_003377999.1**	1105	21501	LOC411477 LOC552476 LOC411476
**NW_003377999.1**	1705343	1788934	LOC100577440 LOC100577887 Rps8 B-gluc1 LOC552341 MED10
**NW_003378009.1**	830749	901643	Vps25 LOC413630 LOC100188940 LOC100579049 Eaat-3 Unc-89
**NW_003378029.1**	1293139	1349024	LOC410024 LOC410023 Appl
**NW_003378036.1**	160294	262584	LOC724585 LOC409360 LOC412458 Spase22-23 Oscillin RpL10Ab LOC724721 Tal
**NW_003378039.1**	2140985	2188370	SP8 LOC726323 Sema-5c Gad1 LOC408510 Tsf1
**NW_003378039.1**	2202418	2245807	LOC726459 Mgat2 LOC552461 LOC408516 LOC552410
**NW_003378045.1**	128258	172938	Trx-2 LOC409452 LOC100578434 LOC100577712
**NW_003378048.1**	681261	708411	LOC100578046 LOC725171 LOC725147 LOC100578864
**NW_003378055.1**	1029216	1044860	LOC100576140 LOC551794 LOC551844
**NW_003378056.1**	57987	93179	LOC726513 Plap Mkp3 LOC552503
**NW_003378062.1**	917531	941608	LOC725310 Atg8a RpL22
**NW_003378065.1**	2302	50118	CYP6AS3 CYP6AS7 Cyp6as5 CYP6AS4
**NW_003378075.1**	879313	968786	LOC100576703 LOC550921 LOC100577643 LOC411171
**NW_003378088.1**	2218959	2274779	LOC727007 LOC552447 LOC727012 LOC410337 LOC552460
**NW_003378088.1**	4512794	4595091	Rpn12 Arp11 LOC100576421 LOC413145
**NW_003378093.1**	451854	499999	LOC410996 Trim9 LOC410999 LOC408784
**NW_003378095.1**	548027	574107	ATF-3-like LOC726721 AlkB
**NW_003378115.1**	458954	533968	LOC724886 Fem Csd
**NW_003378123.1**	2182091	2205050	LOC408669 LOC552073 fat-spondin
**NW_003378123.1**	2704646	2715995	LOC551290 RpS3 Kr-h2 LOC410828
**NW_003378143.1**	211668	260964	CBP LOC412620 nrv2
**NW_003378179.1**	1199905	1248539	sim LOC552014 LOC412734

Supplementary Table 2: Clusters of genes more highly expressed in queen-less worker ovaries as compared with queen-right worker ovaries

Contig	Start	End	Genes
**NW_003377872.1**	127227	150576	Skp2 bor LOC726354 yps
**NW_003377884.1**	307285	346469	LOC727447 LOC552468 LOC413545
**NW_003377899.1**	26605	56161	LOC726735 LOC725270 LOC726812
**NW_003377923.1**	1042854	1102753	LOC551609 CTL6 Neos lqf
**NW_003377938.1**	1773073	1826522	LOC100578129 eIF5B LOC411469
**NW_003377939.1**	1287641	1347844	LOC551090 LOC412308 LOC726537 LOC726586 LOC726482 LOC551462 LOC551545
**NW_003377939.1**	1734052	1815237	JIL-1 LOC100576863 LOC411629 LOC413618 LOC412200

**NW_003377943.1**	199684	316737	Bre1 LOC413376 mip40 LOC412459 ksr
**NW_003377988.1**	289711	488782	Tango13 LOC552192 LOC413341 LOC410940
**NW_003377991.1**	1234979	1259341	LOC408708 LOC725396 disp LOC100579058
**NW_003377999.1**	1026249	1052660	LOC409804 LOC413181 LOC412770
**NW_003377999.1**	2871013	2912289	CycE LOC409009 LOC409010 LOC551265 tacc
**NW_003378010.1**	569549	889360	LOC408373 LOC413660 LOC408374
**NW_003378016.1**	110818	145322	LOC409136 Usp7 LOC727236
**NW_003378021.1**	85636	385007	His2Av LOC408936 LOC409212 LOC551661 rin
**NW_003378026.1**	344614	390948	LOC100576125 LOC724793 LOC411026
**NW_003378026.1**	1015806	1178716	LOC100578579 LOC725208 SF3A1
**NW_003378036.1**	689871	736127	LOC552294 yl FKBP59 LOC552206 LOC725945 LOC725746
**NW_003378039.1**	1012359	1274621	LOC100576569 LOC100576529 cpx
**NW_003378039.1**	3811747	3864107	LOC411646 LOC412828 LOC411410
**NW_003378041.1**	12597	23701	LOC413006 LOC100577382 LOC552151
**NW_003378045.1**	644423	720701	LOC409292 LOC725524 rump RnrL LOC551566 LOC411970
**NW_003378056.1**	533931	612107	LOC413912 eIF3-S8 Vrp1 LOC412150 LOC413652 LOC552031 LOC551928 LOC410037
**NW_003378072.1**	810884	835779	LOC725656 CSN4 porin LOC725783 LOC409483
**NW_003378077.1**	30989	964116	LOC411218 LOC411219 LOC725329 LOC412491
**NW_003378082.1**	762722	808796	LOC726679 lin LOC408693
**NW_003378082.1**	1472095	1506705	LOC411871 LOC409092 Hr78
**NW_003378085.1**	424887	436520	Cap LOC552458 LOC410219
**NW_003378088.1**	384328	435567	LOC413108 kinesin-3B LOC725821 LOC552833
**NW_003378088.1**	2832581	2864895	LOC551735 RnpS1 LOC551683
**NW_003378088.1**	4411349	4483656	hyd bun polo LOC551146 LOC725822 SF3B3 LOC412409
**NW_003378093.1**	1235363	1266830	LOC100576412 Mad toc
**NW_003378095.1**	511189	542332	LOC726991 eIF5 LOC411293
**NW_003378096.1**	609642	643337	LOC408850 LOC100578463 LOC100576817 LOC411891 Rox8
**NW_003378096.1**	2454342	2530706	LOC412707 flfl LOC552581 LOC412593 LOC413451 Mcm5 Pros26.4 LOC100576794 LOC100576413
**NW_003378122.1**	567263	606011	trio LOC410726 LOC725494 LOC100579041
**NW_003378123.1**	457821	506755	LOC409034 LOC550804 LOC725626
**NW_003378123.1**	2779459	2847427	LOC409221 Rtf1 gish LOC725589
**NW_003378125.1**	788093	811878	CDC45L LOC412599 LOC413805
**NW_003378142.1**	621104	650378	LRP6 LOC413195 LOC724439 LOC552223
**NW_003378145.1**	153351	196397	bys LOC100579034 LOC726703 LOC726737 Mcm7
**NW_003378152.1**	48256	145271	MED25 bora LOC409207 LOC100576966 LOC100576908 LOC552439 LOC727246 Rbf ade2
**NW_003378158.1**	895870	977677	Taf11 trc ns4 LOC412986
**NW_003378169.1**	623867	650160	LOC406132 LOC726542 kinesin-5A LOC726468 LOC100577833 LOC726487
**NW_003378177.1**	143107	316988	Sema-1 Nedd4 LOC725609
**NW_003378184.1**	125369	178820	LOC413855 LOC411546 lqfR LOC412745

Supplementary Table 3: Clusters of genes more highly expressed in queen-less worker ovaries (as compared with queen ovaries (Figure 1A), n = 27 genes). Note that there are no clusters of genes detected amongst the 17 genes that are more highly expressed in queen ovaries (as compared with queen-less worker ovaries (Figure 1A), n=17 genes)

Contig	Start	End	Genes	P-value
**NW_003378145.1**	94732	110614	LOC725404 LOC551176 LOC410075	3.27E-03

Supplementary Table 4: Sequencing and mapping statistics for RNA-seq and ChIP-seq data.

Sample	Experiment	Replicate	Sequenced Reads	Mapped Reads	Mapping %
Queen-right worker	RNA-seq	R1	7,160,479	6,984,615	97.54%
		R2	6,964,277	6,803,982	97.70%
Queen-less worker	RNA-seq	R1	7,503,621	7,366,395	98.17%
		R2	7,008,828	6,878,686	98.14%
Queen	RNA-seq	R1	7,192,461	7,066,000	98.24%
		R2	7,196,797	7,064,478	98.16%
Queen-right worker	H3K27me3	R1	49,224,202	41,865,623	85.05%
		R2	46,305,790	38,654,583	83.48%
		Input	50,868,146	39,470,263	77.59%
Queen-less worker	H3K27me3	R1	46,100,192	38,749,832	84.06%
		R2	55,190,016	46,411,815	84.09%
		Input	78,982,855	65395372	82.80%
Queen	H3K27me3	R1	29,967,216	25,845,807	86.25%
		R2	32,415,752	27,970,006	86.29%
		Input	46,341,751	38,152,701	82.33%

Supplementary Table 5: **Clusters of genes associated with differential expression in previously published *A. mellifera* RNA-seq datasets**; two data sets associated with plasticity (Cameron, et al. 2013; Nie, et al. 2018) and three associated with non-plastic physiological changes (Mao, et al. 2013; Corby-Harris, et al. 2014; Pires, et al. 2016).

							Any overlap with honeybee ovary clusters		Strict overlap with honeybee ovary clusters	
		Number of differentially expressed genes	Clusters (gene based)	signficance	Clusters (length based)	significance	gene based	Length based	gene based	Length based
**Cameron et al., 2012**	**Queen vs. worker destined larvae 60h**									
	Queen	1831	38	0.131	42	0.0081	6	12	5	5
	Worker	423	2	0.0171	4	0.099	0	1	0	0
**Corby-Harris et al., 2014**	**Effect of diet and age on transcription in abdomen (minus gut)**									
	Rich Diet vs. Poor Diet									
	Rich	10	0	**1**	0	**1**	0	**0**	0	0
	Poor	0	n/a	**n/a**	n/a	**n/a**	0	0	0	0
	Young vs. old									
	Young	72	0	1	0	1	0	0	0	0
	Old	17	0	1	0	1	0	0	0	0
	Rich Diet vs. Poor diet at 3 days									
	Rich	132	2	0.0016	2	0.0118	1	1	1	0
	Poor	16	0	1	0	1	0	0	0	0
	Rich Diet vs. Poor diet at 8 days									
	Rich	6358	18	0.814	39	0.7503	2	8	0	2
	Poor	2947	31	0.6433	40	0.585	3	9	0	1
	Young vs. Old on a poor diet									
	Young	71	0	1	0	1	0	0	0	0
	Old	13	0	1	0	1	0	0	0	0
	Young vs. Old on a rich diet									
	Young	32	2	0	2	0	1	2	1	0
	Old	21	0	1	0	1	0	0	0	0
**Mao et al., 2013**	**Transcriptional effect of *p*-courmaric acid on midgut**									
	Control	154	0	1	0	1	0	0	0	0
	p-coumaric acid	106	1	0.5316	0	1	0	0	0	0
**Nie et al., 2018**	**Antennae of workers performing different tasks**									
	Forager	1710	49	0.0004	41	0.113	1	1	0	0

	New	1957	42	0.0048	52	0.0289	2	7	0	1
	Forager	228	1	0.0329	1	0.0962	1	1	0	0
	Nurse	152	0	0.0898	1	0.248	0	0	0	0
	Nurse	754	34	0.00008	29	0.0133	4	3	3	2
	New	978	17	0.4026	15	0.7193	1	2	1	0

**Pires et al., 2016**	**Gene expression changes during embryogenesis**									
	Diploid oocyte vs. diploid 2 hrs									
	Oocyte	823	14	0.0025	8	0.542	3	1	1	0
	2 hrs	3385	69	0	76	0	5	20	2	8
	Diploid oocyte vs. diploid 6 hrs									
	Oocyte	488	2	0.169	4	0.17	1	1	0	0
	6 hrs	4256	61	5e−04	83	0	1	19	1	2
	6h vs 18 hrs									
	6 hrs	2369	16	0.7005	16	0.1162	0	3	0	2
	18 hrs	2527	28	0.9904	32	0.0615	3	6	2	3

Supplementary Table 6: Oligonucleotide primer sequences used in this study

Primer	NCBI Gene ID	5’ primer	3’ primer	Fragment size
**Figure 3 Expression of PRC 2 components**				
E(Z)	LOC552235	TCTGCAGAAGATGCTTTAAATACGA	CGCATGGTTCAGCAAAAGGT	117
Eed	LOC551412	CTACCCGCAGTGCTCGTTAT	ACTCCAAACAATGGCTGTCC	136
Su(z)12	LOC409170	AGAAAACCCAGGTGTTCGTG	CAGAAACCTGCATAATTGGTGA	161
Caf1	LOC552200	TCAGTAAAATTGGCGAGGAACA	GGCAGTATGACCACCATGAATAA	87
**Figure S1 Validation of RNA-seq data**				
Ago3	LOC725111	GTAAATTAGGTGGAGCACTTTGG	AAACCAGCTACACTACCTTTCATTG	118
Aub	Aub, LOC412427	GATACCATCAACTCCAGAAAATATACA	GCTTGTAGCATTACATTACTTCCAGT	90
Deltex		Duncan *et al.*, 2016[1]		
Fringe		Duncan *et al.*, 2016[1]		
GB10585 (Her)		Duncan *et al.*, 2016[1]		
Gmap	LOC411348	TGTAAAATGGCGTGGTTCG	TTTGCTTCCTTCAGTGCTTTC	140
Neuralised		Duncan *et al.*, 2016[1]		
Notch		Duncan *et al.*, 2016[1]		
Numb		Duncan *et al.*, 2016[1]		
Serrate		Duncan *et al.*, 2016[1]		
Vg	Vg, LOC406088	GACGTATCCCTGGCTCTGGA	TCGAGTCGTGGTCGAAATTG	103
VgR	yl, LOC725920	ATCGCTGTCGAACCGAGAA	TTGCCCATTGTATTGGATCG	104
**Figure S5 Validation of ChIP-seq data**				
Br-c	Br-c	CTCCATCGCCTGTCATTTTT	CGGGGTAAAGGGGAGAGAG	118
bHLH2	LOC100578067	CTTCTGCAGAGGGTTCCAAG	TCACCGACACCTAGAAGAACC	113
GB10585 (Her)	LOC724465	GACAGTTGGTGGGGGAAAG	TGTTCAGGTCGATTCTCAGC	78
Caf1	LOC552200	TCAGTAAAATTGGCGAGGAACA	GGCAGTATGACCACCATGAATAA	173
Mir79	Mir79	AAAATTCAGTAAGTAAGGTCAAACAAA	TTGTTGTATTCAATATCCACTCAGTT	120
Lethal 7	LOC724488	CTATCGGTAGCCGGTGCTAT	TCAAGCTTCTCTCTCGAGTGTTT	81
Lethal 5	LOC724488	ATTGCGCCTGCTTGTAAAAT	GTTCCACACGGACTGCTGTA	103

[1] Duncan, E.J., Hyink, O., and Dearden, P.K. (2016). Notch signalling mediates reproductive constraint in the adult worker honeybee. Nature Communications *7*.

Supplementary references

Cameron RC, Duncan EJ, Dearden PK. 2013. Biased gene expression in early honeybee larval development. BMC Genomics 14:903.

Cline MS, Smoot M, Cerami E, Kuchinsky A, Landys N, Workman C, Christmas R, Avila-Campilo I, Creech M, Gross B, et al. 2007. Integration of biological networks and gene expression data using Cytoscape. Nature protocols 2:2366.

Corby-Harris V, Jones BM, Walton A, Schwan MR, Anderson KE. 2014. Transcriptional markers of sub-optimal nutrition in developing *Apis mellifera* nurse workers. BMC Genomics 15:134.

Evans KJ, Huang N, Stempor P, Chesney MA, Down TA, Ahringer J. 2016. Stable *Caenorhabditis elegans* chromatin domains separate broadly expressed and developmentally regulated genes. Proceedings of the National Academy of Sciences 113:E7020-E7029.

Huang DW, Sherman BT, Lempicki RA. 2009. Systematic and integrative analysis of large gene lists using DAVID bioinformatics resources. Nature protocols 4:44-57.

Iovino N, Ciabrelli F, Cavalli G. 2013. PRC2 controls *Drosophila* oocyte cell fate by repressing cell cycle genes. Developmental cell 26:431-439.

Mao W, Schuler MA, Berenbaum MR. 2013. Honey constituents up-regulate detoxification and immunity genes in the western honey bee *Apis mellifera*. Proc Natl Acad Sci U S A 110:8842-8846.

Misof B, Liu S, Meusemann K, Peters RS, Donath A, Mayer C, Frandsen PB, Ware J, Flouri T, Beutel RG, et al. 2014. Phylogenomics resolves the timing and pattern of insect evolution. Science 346:763-767.

Nie H, Xu S, Xie C, Geng H, Zhao Y, Li J, Huang WF, Lin Y, Li Z, Su S. 2018. Comparative transcriptome analysis of *Apis mellifera* antennae of workers performing different tasks. Mol Genet Genomics 293:237-248.

Pauler FM, Sloane MA, Huang R, Regha K, Koerner MV, Tamir I, Sommer A, Aszodi A, Jenuwein T, Barlow DP. 2009. H3K27me3 forms BLOCs over silent genes and intergenic regions and specifies a histone banding pattern on a mouse autosomal chromosome. Genome Research 19:221-233.

Pignatelli M, Serras F, Moya A, Guigo R, Corominas M. 2009. CROC: finding chromosomal clusters in eukaryotic genomes. Bioinformatics 25:1552-1553.

Pires CV, Freitas FC, Cristino AS, Dearden PK, Simoes ZL. 2016. Transcriptome Analysis of Honeybee (*Apis Mellifera*) Haploid and Diploid Embryos Reveals Early Zygotic Transcription during Cleavage. PLoS One 11:e0146447.

Prokopuk L, Stringer JM, Hogg K, Elgass KD, Western PS. 2017. PRC2 is required for extensive reorganization of H3K27me3 during epigenetic reprogramming in mouse fetal germ cells. Epigenetics & Chromatin 10:7.

Shen L, Shao NY, Liu X, Maze I, Feng J, Nestler EJ. 2013. diffReps: detecting differential chromatin modification sites from ChIP-seq data with biological replicates. PLoS One 8:e65598.

Simola DF, Graham RJ, Brady CM, Enzmann BL, Desplan C, Ray A, Zwiebel LJ, Bonasio R, Reinberg D, Liebig J. 2016. Epigenetic (re) programming of caste-specific behavior in the ant *Camponotus floridanus*. Science 351:aac6633.

Simola DF, Ye C, Mutti NS, Dolezal K, Bonasio R, Liebig J, Reinberg D, Berger SL. 2013. A chromatin link to caste identity in the carpenter ant *Camponotus floridanus*. Genome Research 23:486-496.

Wilson MJ, Abbott H, Dearden PK. 2011. The evolution of oocyte patterning in insects: multiple cell-signaling pathways are active during honeybee oogenesis and are likely to play a role in axis patterning. Evol Dev 13:127-137.
